# Comparable genetic alteration profiles between gastric cancers with current and past *Helicobacter pylori* infection

**DOI:** 10.1038/s41598-021-02761-7

**Published:** 2021-12-06

**Authors:** Sho Tsuyuki, Hideyuki Takeshima, Shigeki Sekine, Yukinori Yamagata, Takayuki Ando, Satoshi Yamashita, Shin Maeda, Takaki Yoshikawa, Toshikazu Ushijima

**Affiliations:** 1 Division of Epigenomics, National Cancer Center Research Institute, 5-1-1 Tsukiji, Chuo-ku, Tokyo, 104-0045 Japan; 2 Department of Gastroenterology, Yokohama City University Graduate School of Medicine, 3-9 Fukuura, Kanazawa-ku, Yokohama, Kanagawa 236-0004 Japan; 3 Division of Molecular Pathology, National Cancer Center Research Institute, 5-1-1 Tsukiji, Chuo-ku, Tokyo, 104-0045 Japan; 4 Department of Gastric Surgery, National Cancer Center Hospital, 5-1-1 Tsukiji, Chuo-ku, Tokyo, 104-0045 Japan; 5 Third Department of Internal Medicine, University of Toyama, 2630 Sugitani, Toyama, Toyama 930-0194 Japan

**Keywords:** Next-generation sequencing, Gastric cancer, Oncogenes, Cancer genomics, Gastric cancer, Gastric cancer

## Abstract

Gastric cancers can develop even after *Helicobacter pylori* (*H. pylori*) eradication in 0.2–2.9% cases per year. Since *H. pylori* is reported to directly activate or inactivate cancer-related pathways, molecular profiles of gastric cancers with current and past *H. pylori* infection may be different. Here, we aimed to analyze whether profiles of point mutation and gene amplification are different between the two groups. Current or past infection by *H. pylori* was determined by positive or negative amplification of *H. pylori jhpr3* gene by PCR, and past infection was established by the presence of endoscopic atrophy. Among the 90 gastric cancers analyzed, 55 were with current infection, and 35 were with past infection. Target sequencing of 46 cancer-related genes revealed that 47 gastric cancers had 68 point mutations of 15 different genes, such as *TP53* (36%), *KRAS* (4%), and *PIK3CA* (4%) and that gene amplification was present for *ERBB2*, *KRAS*, *PIK3CA*, and *MET* among the 26 genes assessed for copy number alterations. Gastric cancers with current and past infection had similar frequencies of *TP53* mutations (38% and 31%, respectively; *p* = 0.652) and oncogene activation (20% and 29%, respectively; *p* = 0.444). Gastric cancers with current and past infection had comparable profiles of genetic alterations.

## Introduction

*Helicobacter pylori* (*H. pylori*) is almost the exclusive cause of gastric cancers^1,2^, and *H. pylori*-triggered chronic inflammation is deeply involved in gastric carcinogenesis^3–6^. At the molecular level, aberrant DNA methylation is strongly induced by *H. pylori* infection-triggered chronic inflammation long before cancer development^7,8^. Aberrant DNA methylation of promoter CpG islands can suppress various tumor-suppressor genes, such as *CDKN2A* encoding p16 and *CDH1* encoding E-cadherin^9,10^. Genetic alterations are also induced by *H. pylori* infection-triggered inflammation. Up-regulated *AID*, which encodes cytidine deaminase, and accumulation of *TP53* mutation in gastric mucosa inflamed by *H. pylori* is well known^11^. Accumulation of both epigenetic and genetic alterations in gastric mucosa is associated with increased cancer risk, forming a field for cancerization^12^.

Despite the presence of the field, *H. pylori* eradication has the benefit of preventing gastric cancers^13–16^. Eradication therapy has been covered by public health insurance since 2013 in Japan, and 1,400,000 or more healthy people with *H. pylori* infection are treated with the therapy every year^17^. However, even after *H. pylor*i eradication, gastric cancer develops at an incidence of 0.15–0.67% per year in healthy individuals^18^, and metachronous gastric cancers develop at an incidence of 1.4–2.9% per year in gastric cancer patients who underwent endoscopic submucosal dissection^14,19,20^. The presence of a field for cancerization suggests that molecular profiles in gastric cancers with current and past *H. pylori* infection are the same. At the same time, *H. pylori* itself can enhance pro-oncogenic signaling pathways involved in the proliferation and differentiation of cells, mainly mediated by CagA^1,3^. This suggests the possibility that different signaling pathways can be active between gastric cancers with current and past *H. pylori* infection.

Here, we aimed to analyze whether genetic alterations, namely point mutations and gene amplifications, are the same or different between gastric cancers with current and past *H. pylori* infection.

## Results

### 52% of all cancers had somatic point mutations of cancer-related genes

**Figure 1:**
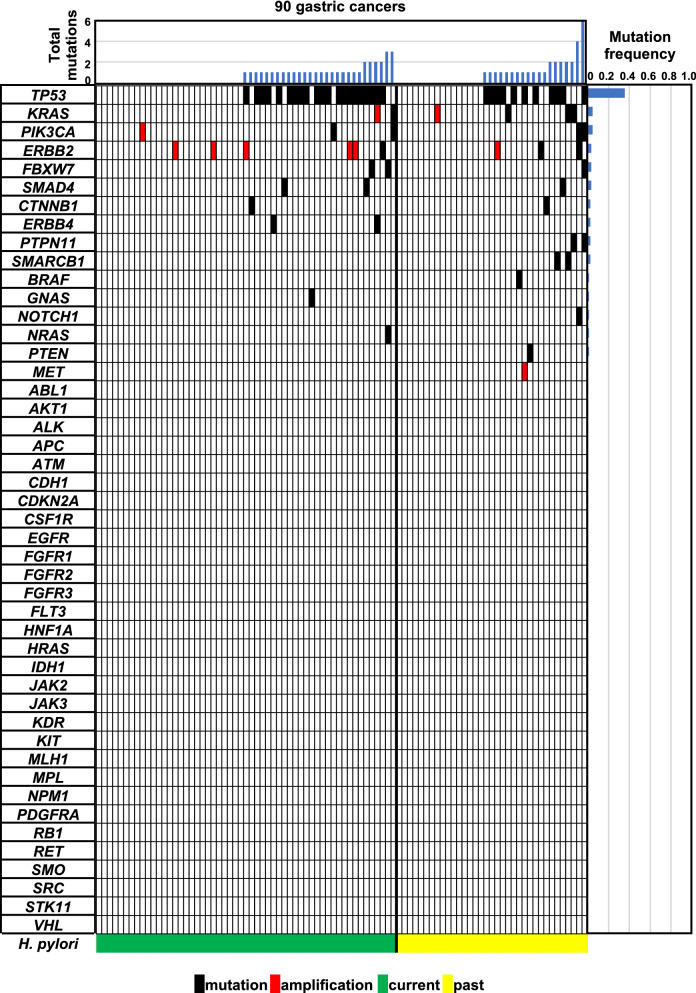
Profiles of genetic alterations in 90 gastric cancers. Genetic alterations of 46 cancer-related genes were analyzed by next-generation target sequencing. Among the 90 gastric cancers, 47 cancers had 68 somatic point mutations of 15 different genes, such as *TP53*, *KRAS*, and *PIK3CA*. Ten cancers had gene amplification of one of *ERBB2*, *KRAS*, *PIK3CA*, and *MET*. Gastric cancers in individual groups and genes analyzed were aligned in the order of the number of total mutations and mutation frequency, respectively. Black and red boxes show somatic point mutations and gene amplifications, respectively. Gastric cancers with current and past infection had comparable profiles of somatic point mutations and gene amplifications.

Among the 90 gastric cancers (Supplementary Figure S1), current *H. pylori* infection was detected in 55 cancers, and 35 cancers were considered to have had past infection. Target sequencing of 46 cancer-related genes was conducted for the 90 gastric cancers, and 47 cancers (52%) had 68 somatic point mutations of 15 different genes (*TP53*, *KRAS*, *PIK3CA*, *ERBB2*, *FBXW7*, *SMAD4*, *CTNNB1*, *ERBB4*, *PTPN11*, *SMARCB1*, *BRAF*, *GNAS*, *NOTCH1*, *NRAS,* and *PTEN*) (Tables 1 and 2). Among the 68 mutations, 66 were missense mutations and 2 were nonsense mutations. *TP53* was most frequently mutated (32 of the 90 gastric cancers, 36%). *KRAS*, *PIK3CA*, *ERBB2*, *FBXW7*, *SMAD4*, *CTNNB1*, *ERBB4*, *PTPN11*, and *SMARCB1* were mutated in multiple gastric cancers (Fig. 1). 5, 5, 3, and 2 mutations of *KRAS*, *PIK3CA*, *ERBB2* and *CTNNB1* were observed in 4, 4, 3, and 2 gastric cancers, respectively (5, 3, 3, and 2 hotspot mutations, respectively) (Tables 1 and 2). These results showed that 12% of gastric cancers had activating point mutations of oncogenes (Table 3).

**Table 1:** List of somatic mutations in the 55 gastric cancers with current *H. pylori* infection.

Sample	Gene	Coverage	Variant allele frequency	Nucleotide change	Amino acid change
B-GC1	*TP53*	3157	30.6	c.818G>A	p.Arg273His
B-GC3	*SMAD4*	9192	13.2	c.1525T>G	p.Trp509Gly
	*TP53*	4942	30.3	c.857A>T	p.Glu286Val
B-GC8	No mutation				
B-GC11	No mutation				
B-GC12	No mutation				
B-GC14	No mutation				
B-GC15	*CTNNB1*^a^	4389	79.7	c.101G>A	p.Gly34Glu
B-GC16	*TP53*	5638	31.6	c.818G>A	p.Arg273His
B-GC17	*NRAS*	5643	29.2	c.34G>T	p.Gly12Cys
	*FBXW7*	6577	28.4	c.1514G>A	p.Arg505His
	*FBXW7*	4646	21.9	c.1394G>A	p.Arg465His
B-GC19	*TP53*	6252	14.8	c.818G>A	p.Arg273His
B-GC22	*TP53*	5911	38.0	c.844C>T	p.Arg282Trp
B-GC23	No mutation				
B-GC27	*ERBB4*	9636	25.1	c.1817A>G	p.Lys606Arg
B-GC33	*TP53*	4987	63.1	c.743G>A	p.Arg248Gln
B-GC34	No mutation				
B-GC35	No mutation				
B-GC37	No mutation				
B-GC38	No mutation				
B-GC39	No mutation				
B-GC52	No mutation				
B-GC56	No mutation				
B-GC63	*SMAD4*	1269	34.8	c.1082G>A	p.Arg361His
B-GC64	No mutation				
B-GC66	*PIK3CA*^a^	618	15.7	c.1624G>A	p.Glu542Lys
	*KRAS*^a,b^	336	22.3	c.34G>A	p.Gly12Ser
	*KRAS*^a,b^	333	18.0	c.35G>A	p.Gly12Asp
B-GC70	No mutation				
B-GC71	*TP53*	1066	66.8	c.659A>G	p.Tyr220Cys
B-GC73	No mutation				
B-GC74	*TP53*	1520	55.6	c.853G>A	p.Glu285Lys
B-GC75	*TP53*	602	51.3	c.536A>G	p.His179Arg
B-GC77	*TP53*	566	43.8	c.404G>A	p.Cys135Tyr
B-GC80	*TP53*	546	43.4	c.536A>G	p.His179Arg
	*FBXW7*	1063	46.8	c.1393C>T	p.Arg465Cys
B-GC81	No mutation				
B-GC83	*GNAS*	222	12.6	c.2531G>A	p.Arg844His
B-GC85	No mutation				
B-GC86	*TP53*	1664	59.1	c.818G>T	p.Arg273Leu
B-GC87	*TP53*	569	31.6	c.388C>G	p.Leu130Val
B-GC88	*TP53*	675	52.6	c.524G>A	p.Arg175His
	*ERBB4*	988	53.7	c.719G>A	p.Gly240Glu
B-GC90	*TP53*	1833	20.7	c.818G>A	p.Arg273His
B-GC92	No mutation				
B-GC95	No mutation				
B-GC96	*PIK3CA*^a^	806	12.3	c.1633G>A	p.Glu545Lys
B-GC97	No mutation				
B-GC98	No mutation				
S2	*TP53*	496	34.1	c.581T>G	p.Leu194Arg
S4	*TP53*	438	74.2	c.581T>G	p.Leu194Arg
S13	*TP53*	70	15.7	c.478A>G	p.Met160Val
	*ERBB2*^a^	482	23.9	c.2264T>C	p.Leu755Ser
S17	No mutation				
S19	No mutation				
S20	No mutation				
S21	No mutation				
S22	No mutation				
S23	*TP53*	565	67.8	c.537T>A	p.His179Gln
S36	*TP53*	1142	34.9	c.524G>A	p.Arg175His
S43	*TP53*	239	74.9	c.1024C>T	p.Arg342Ter
S124	No mutation				

^a^Activated oncogene mutation.

^b^These mutations did not exist on the same allele.

**Table 2:** List of somatic mutations in the 35 gastric cancers with past *H. pylori* infection.

Sample	Gene	Coverage	Variant allele frequency	Nucleotide change	Amino acid change
B-GC2	No mutation				
B-GC6	*TP53*	504	22.4	c.524G>A	p.Arg175His
B-GC9	No mutation				
B-GC13	*TP53*	4255	21.1	c.380C>T	p.Ser127Phe
	*TP53*	5126	18.7	c.376T>C	p.Tyr126His
B-GC18	No mutation				
B-GC21	No mutation				
B-GC25	*TP53*	3216	13.0	c.535C>T	p.His179Tyr
B-GC26	No mutation				
B-GC28	No mutation				
B-GC30	No mutation				
B-GC41	*ERBB2*^a^	4450	42.9	c.2434G>A	p.Val812Ile
	*NOTCH1*	5375	28.5	c.4723G>C	p.Val1575Leu
	*PIK3CA*	2109	32.5	c.1031T>G	p.Val344Gly
	*PIK3CA*	3448	12.2	c.2091G>A	p.Met697Ile
B-GC43	No mutation				
B-GC45	*TP53*	5152	85.9	c.814G>A	p.Val272Met
B-GC46	No mutation				
B-GC47	No mutation				
B-GC50	No mutation				
B-GC51	No mutation				
B-GC53	No mutation				
B-GC55	*TP53*	3195	42.7	c.637C>T	p.Arg213Ter
B-GC58	*KRAS*^a^	664	28.2	c.35G>A	p.Gly12Asp
B-GC60	No mutation				
B-GC61	*TP53*	1025	24.8	c.742C>T	p.Arg248Trp
	*TP53*	1263	30.8	c.565G>A	p.Ala189Thr
	*TP53*	649	25.3	c.523C>T	p.Arg175Cys
	*PTPN11*	1566	31.0	c.214G>A	p.Ala72Thr
	*FBXW7*	844	30.0	c.1393C>T	p.Arg465Cys
	*PIK3CA*^a^	337	23.7	c.3140A>G	p.His1047Arg
B-GC62	*TP53*	1062	25.6	c.659A>G	p.Tyr220Cys
B-GC68	*BRAF*	1593	13.1	c.1406G>C	p.Gly469Ala
B-GC72	*SMAD4*	1347	10.3	c.1081C>T	p.Arg361Cys
	*TP53*	1290	26.7	c.536A>G	p.His179Arg
B-GC78	*TP53*	1057	24.1	c.818G>A	p.Arg273His
	*SMARCB1*	603	21.9	c.1129C>T	p.Arg377Cys
B-GC82	No mutation				
B-GC91	*TP53*	1305	37.2	c.844C>T	p.Arg282Trp
B-GC99	*PTEN*	1370	27.2	c.752G>T	p.Gly251Val
S5	*KRAS*^a^	1626	54.4	c.38G>A	p.Gly13Asp
	*SMARCB1*	50	56	c.1130G>A	p.Arg377His
S6	*TP53*	2077	24.7	c.820G>C	p.Val274Leu
S12	*ERBB2*^a^	24,516	63.8	c.2264T>C	p.Leu755Ser
S31	*KRAS*^a^	1979	56.6	c.35G>T	p.Gly12Val
	*PTPN11*	7391	56.8	c.182A>G	p.Asp61Gly
S40	No mutation				
S47	*CTNNB1*^a^	4591	33.7	c.121A>G	p.Thr41Ala

^a^Activated oncogene mutation.

**Table 3:** Molecular profiles in 90 gastric cancers.

Characteristic	*H. pylori* infection status		*p* value
	Current	Past	
	N (%)	N (%)	
**Oncogene point mutations in hotspots**			
Yes	4 (7.3)	7 (20.0)	0.100
No	51 (92.7)	28 (80.0)	
**Gene amplification of oncogenes**			
Yes	7 (12.7)	3 (8.6)	0.735
No	48 (87.3)	32 (91.4)	
**Oncogene activation (either or both point mutations in hotspots and gene amplification)**			
Yes	11 (20.0)	10 (28.6)	0.444
No	44 (80.0)	25 (71.4)	

Regarding SNPs observed in gastric cancer patients, their frequencies were compared between gastric cancer patients and healthy Japanese people in datasets of the Tohoku Medical Megabank Organization (ToMMo 4.7 K JPN). SNPs of *PIK3CA* (p.Glu707Lys) and *KDR* (p.Gln472His) were more frequent in gastric cancer patients than in healthy Japanese people (*p* < 2.2 × 10^–16^ and *p* = 0.001, respectively; Bonferroni-corrected significance level = 0.003) (Supplementary Table S1). Pathogenicity of these SNPs was assessed using the Catalogue of Somatic Mutations in Cancer (COSMIC) database. *PIK3CA* (p.Glu707Lys) and *KDR* (p.Gln472His) were registered as “Pathogenic” and “Neutral”, respectively. Therefore, *PIK3CA* (p.Glu707Lys) could be a germline mutation that confers tumor predisposition. 16 other SNPs did not give Bonferroni-corrected statistical significance considering multiple testing.

### *ERBB2*, *KRAS*, *PIK3CA*, and *MET* were amplified

**Figure 2:**
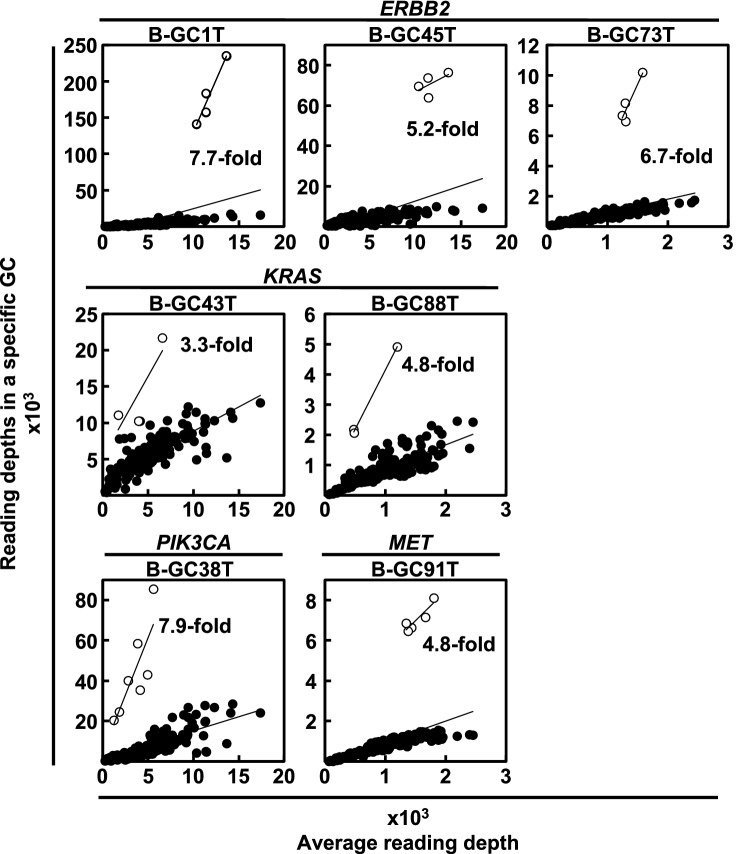
Gene amplification analysis of cancer-related genes. Gene amplification of 26 cancer-related genes was evaluated by utilizing reading depth of individual genes. For an individual sample, reading depths of 160 amplicons were plotted in a panel. Each amplicon was expected to be on a regression line calculated from all amplicons, but amplicons of the amplified gene were outlying. *ERBB2* was amplified in 3 gastric cancers; *KRAS* was amplified in 2 cancers; and *PIK3CA* and *MET* were amplified in one cancer. Open circles show the amplicon of amplified genes. Black circles show that of all the other genes.

Gene amplifications were analyzed for 26 cancer-related genes. Among the 90 gastric cancers, 10 cancers had gene amplification of one of *ERBB2*, *KRAS*, *PIK3CA*, and *MET* (Figs. 1, 2, Supplementary Table S2). *ERBB2* was most frequently amplified (6 of the 90 gastric cancers, 7%), and *KRAS* (2 cancers, 2%), *PIK3CA* (1 cancer, 1%), and *MET* (1 cancer, 1%) followed. Combined with somatic hotspot mutations, *ERBB2* was activated in 9 of the 90 gastric cancers (10%), *KRAS* was in 6 (7%), and *PIK3CA* was in 4 (4%). These results showed that 23% of gastric cancers had genetic activation of known oncogenes (Table 3).

### Molecular profiles were similar between gastric cancers with current and past *H. pylori* infection

**Figure 3:**
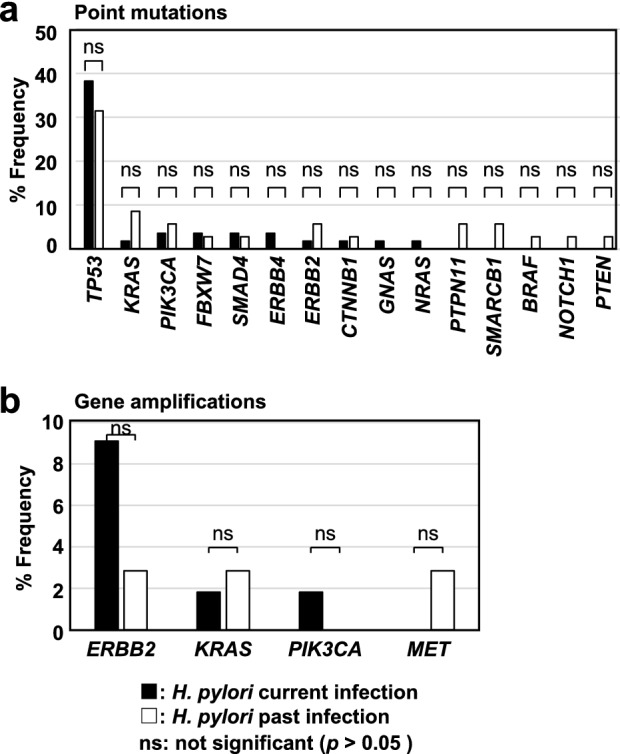
Frequency of point mutations and gene amplification in gastric cancers with current and past *H. pylori* infection. (**a**) Frequency of point mutations. Mutation frequencies of *TP53*, *KRAS*, and *PIK3CA* were similar between gastric cancers with current *H. pylori* infection (*TP53*, 38%; *KRAS*, 2%; and *PIK3CA*, 4%) and those with past infection (*TP53*, 31%; *KRAS*, 9%; and *PIK3CA*, 6%). Black and white bars show frequencies in gastric cancers with current and past *H. pylori* infection, respectively. (**b**) Frequency of gene amplification of *ERBB2*, *KRAS*, *PIK3CA*, and *MET*. The frequency was similar between gastric cancers with current *H. pylori* infection (*ERBB2*, 9%; and *KRAS*, 2%) and those with past infection (*ERBB2*, 3%; and *KRAS*, 3%). Black and white bars show frequencies in gastric cancers with current and past *H. pylori* infection, respectively.

To analyze whether molecular profiles between gastric cancers with current and past *H. pylori* infection are different, frequencies of the somatic point mutations and gene amplifications were compared between the two groups. Both groups had similar frequencies of *TP53* mutations (38% and 31% in gastric cancers with current and past infection, respectively; *p* = 0.652), *KRAS* mutations (2% and 9%; *p* = 0.295), and *PIK3CA* mutations (4% and 6%; *p* = 0.641) (Fig. 3a). As for gene amplifications, gastric cancers with current and past infection also had similar frequencies of *ERBB2* amplification (9% and 3%; *p* = 0.398) and *KRAS* amplification (2% and 3%; *p* = 1.000) (Fig. 3b, Table 3). These results showed that gastric cancers with current and past infection had comparable profiles of genetic alterations.

## Discussion

Gastric cancers with current and past *H. pylori* infection had comparable profiles of genetic alterations, namely somatic point mutations and gene amplification. Even when activation of known oncogenes, such as *ERBB2* and *PIK3CA*, by either a point mutation or gene amplification was analyzed, both groups had similar frequencies. Since genetic activation of these genes has been clinically utilized in molecular targeted therapy^21,22^, it was considered that similar therapeutic strategies can be applicable for both gastric cancers with current and past infection.

It is known that *H. pylori* can directly activate oncogenic pathways, such as the MEK-ERK pathway and WNT pathway, and inactivate tumor-suppressive pathways, such as the p53 pathway, by injecting CagA into epithelial cells^3^. Therefore, it was considered that the alteration mechanisms of cancer-related signaling pathways might be different between gastric cancers with current infection and those with past infection. However, both groups had similar frequencies of alterations of genes involved in these cancer-related pathways. This suggested that direct activation or inactivation of cancer-related pathways by *H. pylori* has limited influence on genetic alterations.

Approximately 47% and 46% of gastric cancers with current *H. pylori* infection and past infection, respectively, had no genetic alterations of known cancer-related genes. In such gastric cancers, repression of tumor-suppressive pathways, such as cell cycle regulation and the p53 pathway, and activation of oncogenic pathways, such as the WNT pathway, are known to be frequently caused by epigenetic alterations, namely aberrant DNA methylation^23^. Therefore, it was considered that epigenetic alterations might be important in both gastric cancers with current *H. pylori* infection and past infection.

Somatic point mutations were analyzed by next-generation target sequencing, which covered 190 regions of 46 cancer-related genes. Although this panel covered almost all of the mutation hot spots of oncogenes, such as *KRAS*, *PIK3CA*, and *CTNNB1*, it covered limited regions of tumor-suppressor genes, such as *TP53* (55.3%), *CDH1* (7.5%), and *MLH1* (2.6%). In addition, this panel did not cover several genes known to be mutated in 10% or more of gastric cancers, such as *ARID1A*, *CREBBP*, *ERBB3*, *SMARCA4*, and *TGFBR2*. Gene amplification was analyzed for 26 genes, including both oncogenes and tumor-suppressor genes, but was detected only in oncogenes, supporting the methodological validity. Approximately 9% of gastric cancers are known to be affected by Epstein-Barr virus (EBV), but EBV infection status was not analyzed in this study. EBV-positive gastric cancers are reported to have recurrent mutations of *PIK3CA*, *ARID1A*, and *BCOR* and amplifications of *JAK2*, *PD-L1*, and *PD-L2*^24^.

Eradication of *H*. *pylori* is known to prevent the progression of gastric atrophy or intestinal metaplasia (IM)^25^, and almost all patients with gastric cancers are known to have gastric atrophy or IM. Actually, also in this study, most patients with past *H*. *pylori* infection had atrophy (Supplementary Figure S1). Although information on clinical history will improve the data quality, we consider that the number of patients with *H. pylori* eradicated before the development of gastric atrophy or IM would be small.

In conclusion, gastric cancers with current *H. pylori* infection and those with past infection had comparable profiles of genetic alterations.

## Methods

### Clinical samples

Surgically resected and fresh-frozen samples of 96 pairs of gastric cancers and corresponding non-cancerous tissues were obtained from the National Cancer Center Biobank. Twenty-one pairs of gastric cancers and corresponding non-cancerous tissues were collected for our previous study^23^, and also used for this study. This study was approved by the Institutional Review Boards of the National Cancer Center, Japan (2012-305 and 2018-024), and written informed consents were obtained from all the patients. All methods were carried out in accordance with relevant guidelines and regulations. Genomic DNA was extracted from gastric cancers and corresponding non-cancerous tissues by the phenol/chloroform method.

### Analysis of *H. pylori* infection status

The infection status of *H. pylori* was determined by detection of PCR products specific for *H. pylori jhpr3* gene and endoscopic gastric atrophy. Sensitivity and specificity for *H*. *pylori* detection by PCR test, urea breath test and serology test are reported to be > 95% and > 95%, 95.9% and 95.7%, and 76–84% and 79–90%, respectively^26^. Therefore, the reliability of a PCR test can be considered to be comparable with the other two methods. To avoid false-negative results in PCR, the quality of genomic DNA extracted from non-cancerous tissues was first evaluated by measuring the copy number of *RPPH1* using quantitative PCR (qPCR) with primers listed in Supplementary Table S3^27^. Among the 117 samples, 110 samples had 1,000 copies or more in 10 ng of genomic DNA, and were qualified for the evaluation of *H. pylori* infection status.

The presence of *H. pylori* was evaluated by qPCR using primers specific to the *jhpr3* gene of *H. pylori*^8^ (Supplementary Table S4) and 100 ng of genomic DNA from non-cancerous tissues. Samples with successful amplification of the *jhpr3* gene in two independent experiments were regarded as *H. pylori*-positive, and those in neither experiment were regarded as negative. Samples with one positive and one negative result were excluded from the entire analysis. Among the 110 samples, 59 samples were *H. pylori*-positive, 36 samples were -negative, and 15 samples were excluded. Endoscopic gastric atrophy was evaluated according to the endoscopic atrophic-border scale described by Kimura and Takemoto^28^. Fifty-seven of 59 *H. pylori*-positive samples had gastric atrophy (current infection), and 35 of 36 *H. pylori*-negative samples had gastric atrophy (past infection). These 92 samples (57 samples with current infection and 35 samples with past infection) were used for next-generation target sequencing. Clinicopathological characteristics, sex and pathology classification were not different among the two groups, but patients with past infection were slightly older (*p* = 0.033) (Supplementary Table S5).

### Next-generation target sequencing

Next-generation target sequencing was conducted using an Ion AmpliSeq Cancer Panel Kit (Thermo Fisher Scientific, Waltham, MA), as described previously^23,29^. The sequence library was prepared by a multiplex PCR, which amplified 190 regions of 46 cancer-related genes. The library DNA was loaded onto an Ion PI Chip v3 (Thermo Fischer Scientific) or Ion 318 Chip v2 (Thermo Fischer Scientific) using Ion Chef (Thermo Fischer Scientific), and was sequenced using an Ion Proton sequencer (Thermo Fischer Scientific) or an Ion PGM sequencer (Thermo Fischer Scientific). The sequences obtained were mapped onto the human reference genome hg19 with Torrent Suite 5.0 (Thermo Fischer Scientific). An amplicon with 50 reads or less was considered to have low coverage, and two samples with 10% or more amplicons of low coverage were excluded from the analysis. Finally, 55 samples with current infection and 35 samples with past infection were used for mutation and amplification analysis. A variant call was conducted using CLC Genomics Workbench 20.0 (Qiagen, Hilden, Germany) with the following criteria; (i) with an allele frequency of 10% or more, (ii) not in homopolymers with 3 bp or more, (iii) present in both forward and reverse reads, and (iv) with a non-synonymous amino acid change. Sequence variations registered in dbSNP Build 137 were considered as SNPs, and were excluded before Sanger sequencing.

### Sanger sequencing

Genomic regions where a sequence variation was found were amplified using 20 ng of genomic DNA (gastric cancers and corresponding non-cancerous tissues) and primers listed in Supplementary Table S6. The PCR products were purified by a DNA Clean and Concentrator-5 Kit (Zymo Research, Irvine, CA), and were sequenced by using a BigDye Terminator v3.1 Cycle Sequencing Kit (Thermo Fischer Scientific) and 3730xl DNA Analyzer (Thermo Fischer Scientific). Sequence variations detected only in gastric cancers were considered as a somatic point mutation. Hotspot mutations were defined using information registered in COSMIC. Namely, a pathogenic mutation at the specific base position whose frequency was 5% or more of all the mutations in a specific gene was defined as a hotspot mutation. Among the 154 variations detected in 72 gastric cancers (newly analyzed cases in this study) by a next-generation sequencer, 101 variations were confirmed by Sanger sequencing (54 and 47 were somatic mutations and SNPs, respectively).

### Analysis of SNPs

Six sequence variations registered in dbSNP Build 137 and twelve sequence variations confirmed as a SNP by Sanger sequencing were considered as SNPs (Supplementary Table S1). The frequencies of identified SNPs in gastric cancer patients (cases in this study) and healthy Japanese people in datasets of the Tohoku Medical Megabank Organization (ToMMo 4.7K JPN) were compared by the Fisher’s exact test.

### Analysis of gene amplification

Gene amplification was analyzed using a next-generation sequencer since copy number variations (CNVs) detected by next-generation sequencers are now known to be accurately confirmed by Multiplex ligation-dependent probe amplification (MLPA), the gold-standard method to evaluate CNVs (Specificity 100%)^30^. Gene amplification of 26 genes (*ABL1*, *APC*, *ATM*, *CDH1*, *EGFR*, *ERBB2*, *ERBB4*, *FBXW7*, *FGFR2*, *FGFR3*, *FLT3*, *KDR*, *KIT*, *KRAS*, *MET*, *PDGFRA*, *PIK3CA*, *PTEN*, *RB1*, *RET*, *SMAD4*, *SMARCB1*, *SMO*, *STK11*, *TP53,* and *VHL*), which had three PCR amplicons or more, was analyzed, as described previously^23^. For an individual sample, reading depths of 160 amplicons of the 26 genes in the sample (y-axis) and in all the samples (average, x-axis) were plotted. The amplicons were expected to be on a regression line, but amplicons of an amplified gene were outlying. The ratio of the slope of a specific gene to that of the all genes was calculated, and genes with a ratio of three or more were defined as amplified genes. Since the next-generation target sequencing of 74 gastric cancers newly collected in this study was conducted in two sequencing runs, there were two background average reading depths (Supplementary Tables S7 and S8). The origins of gastric cancer samples with gene amplification (from our previous study or new in this study) are noted (Supplementary Table S2).

### Statistical analysis

The Fisher’s exact test was used to analyze categorical variables and the Mann–Whitney U test was used to analyze quantitative variables. *p* < 0.05 was considered to be statistically significant.

## Supplementary Information

Supplementary Information 1.

Supplementary Information 2.

## Data Availability

The data that support the findings of this study are available from the corresponding author upon reasonable request.
