# Supplementary material for: Comparable genetic alteration profiles between gastric cancers with current and past Helicobacter pylori infection
Source: Sci Rep. 2021 Dec 6;11:23443. doi: 10.1038/s41598-021-02761-7 (PMC8648804; doi:10.1038/s41598-021-02761-7)
Supplement: Supplementary file 1 — Supplementary Information 1. [file 41598_2021_2761_MOESM1_ESM.pdf]

Comparable genetic alteration profiles between gastric cancers with current and past *Helicobacter pylori* infection

Sho Tsuyuki, Hideyuki Takeshima, Shigeki Sekine, Yukinori Yamagata, Takayuki Ando,
Satoshi Yamashita, Shin Maeda, Takaki Yoshikawa, and Toshikazu Ushijima

Supplementary Table S1. Frequency of SNPs

SNP/dbSNP	gene	Chromosome	Region	Type	Nucleotide change	Amino acid change	Cases in this study N (Frequency)	Cases in ToMMo 4.7K JPN N (Frequency)	*p* value
SNP	*PIK3CA*	chr3	178938877	SNV	c.2119G>A	p.Glu707Lys	36 (0.500)	0 (0)	$< 2.2 \times 10^{-16}$
	TP53	chr17	7578283	SNV	c.566C>T	p.Ala189Val	1 (0.014)	0 (0)	0.007
	ATM	chr11	108236200	SNV	c.9136A>G	p.Ser3046Gly	1 (0.014)	1 (1.05×10^{-4})	0.015
	KDR	chr4	55980324	SNV	c.767T>C	p.Ile256Thr	1 (0.014)	5 (5.24×10^{-4})	0.044
	MLH1	chr3	37067242	SNV	c.1153C>T	p.Arg385Cys	1 (0.014)	7 (7.33×10^{-4})	0.058
	MET	chr7	116411986	SNV	c.3025C>T	p.Pro1009Ser	1 (0.014)	7 (7.33×10^{-4})	0.058
	KDR	chr4	55946163	SNV	c.4016C>T	p.Thr1339Ile	1 (0.014)	12 (1.26×10^{-3})	0.093
	MET	chr7	116340263	SNV	c.1125C>G	p.Asn375Lys	1 (0.014)	13 (1.36×10^{-3})	0.100
	NOTCH1	chr9	139399426	SNV	c.4717A>G	p.Thr1573Ala	1 (0.014)	13 (1.36×10^{-3})	0.100
	KIT	chr4	55593461	SNV	c.1618G>C	p.Val540Leu	1 (0.014)	41 (4.29×10^{-3})	0.271
	TP53	chr17	7579882	SNV	c.31G>C	p.Glu11Gln	1 (0.014)	67 (7.02×10^{-3})	0.401
	CDKN2A	chr9	21971161	SNV	c.197A>G	p.His66Arg	1 (0.014)	82 (8.60×10^{-3})	0.466
dbSNP	*KDR*	chr4	55972974	SNV	c.1416A>T	p.Gln472His	43 (0.597)	3819 (0.400)	0.001
	JAK3	chr19	17945696	SNV	c.2164G>A	p.Val722Ile	1 (0.014)	0 (0)	0.007
	STK11	chr19	1223125	SNV	c.1062C>G	p.Phe354Leu	6 (0.083)	407 (0.043)	0.130
	KIT	chr4	55593464	SNV	c.1621A>C	p.Met541Leu	5 (0.069)	431 (0.045)	0.262
	MLH1	chr3	37067240	SNV	c.1151T>A	p.Val384Asp	4 (0.056)	304 (0.032)	0.294
	MET	chr7	116340262	SNV	c.1124A>G	p.Asn375Ser	4 (0.056)	353 (0.037)	0.343

Supplementary Table S2. The list of samples with gene amplification.

Infection status	Sample	Gene	Fold change	Reference
Current	B-GC1	*ERBB2*	7.7	this study
	B-GC38	*PIK3CA*	7.9	this study
	B-GC73	*ERBB2*	6.7	this study
	B-GC88	*KRAS*	4.8	this study
	S17	*ERBB2*	3.6	[23]
	S23	*ERBB2*	10.5	[23]
	S36	*ERBB2*	5.4	[23]
Past	B-GC43	*KRAS*	3.3	this study
	B-GC45	*ERBB2*	5.2	this study
	B-GC91	*MET*	4.8	this study

Supplementary Table S3. The primer for quality check of DNA

Gene	Forward Primer (5' -> 3')	Reverse primer (5' -> 3')	Amplified region	Amplicon length(bp)	Reference
RPPH1	GGAGGGAAGCTCATCAGTGG	CGTTCTCTGGGAACTCACCT	chr14:20811419-20811563	145	[27]

Supplementary Table S4. The primer for detection of *H. pylori*

Gene	GenBank number	Forward Primer (5' -> 3')	Reverse primer (5' -> 3')	Amplicon length(bp)	Reference
jhpr3	NC000921	AAGGATACTGCCTCCGTAAG	ACAATCCGAACTGAGAGGTG	158	[8]

Supplementary Table S5. Baseline characteristics of 90 patients

Characteristic	*H. pylori* infection status		*p* value
	Current group (N = 55)	Past group (N = 35)	
Age at surgery, mean (SD)			0.033
	67.8 (11.7)	73.4 (9.1)	
Gender, N (%)			0.267
Male	38 (69.1)	20 (57.1)	
Female	17 (30.9)	15 (42.9)	
Pathology of GC, N (%)			0.394
Differentiated type	29 (52.7)	15 (42.9)	
pap	2 (3.6)	2	
tub	27 (49.1)	13	
Undifferentiated type	26 (47.3)	20 (57.1)	
por	24 (43.6)	18 (51.4)	
sig	1 (2.0)	0 (0)	
muc	1 (2.0)	2 (5.7)	

Supplementary Table S6. Primers used for dideoxy sequencing

Gene	Target variation	Forward Primer (5' -> 3')	Reverse primer (5' -> 3')	Amplified region	Amplicon length(bp)	Reference
ABL1	c.1107del	CGTGTTGAAGTCCTCGTTGT	CCCCTACCTGTGGATGAAGTTT	chr9:133748216-133748431	216	This study
ABL1	c.1131_1132insG	ATCTCGTCAGCCATGGAGTA	AATGTGTTGCCAGCACTGAG	chr9:133748378-133748551	174	This study
ABL1	c.1281delC	GGTAGCTGATTTTGGCCTGA	GGAAGAGCAAGAAAGAGGCA	chr9:133750303-133750515	213	This study
ATM	c.9136A>G	GAAAGGAGTGGAAGAAGGCA	AGAGTGAAAGCAGAGATGTTCC	chr11:108236115-108236438	324	This study
BRAF	c.1406G>C	ACGGGACTCGAGTGATGATT	GCGAACAGTGAATATTTCCTTTGA	chr7:140481274-140481479	206	This study
CDH1	c.1203_1204insC	AATCCTTTAGCCCCCTGAGA	TCACTGGATTTGTGGTGACG	chr16:68847139-68847369	231	This study
CDH1	c.228delA	TCTTCCCACAAGTTCGCTCT	AATGTCAACGGTACCAAGGC	chr16:68835486-68835881	396	This study
CDKN2A	c.197A>G	ACACAAGCTTCCTTTCCGTC	ACCAGCGTGTCCAGGAAG	chr9:21971074-21971273	200	This study
CTNNB1	c.101G>A	GCTGATTTGATGGAGTTGGAC	TTGGGAGGTATCCACATCCT	chr3:41266016-41266186	171	This study
EGFR	c.2506delC	GAATTCGGATGCAGAGCTTC	ATTCTTTCTCTTCCGCACCC	chr7:55259346-55259547	202	This study
ERBB2	c.2434G>A	GCAAACCCCTATGTCCACAA	TCTGCATGGTACTCTGTCTCGT	chr17:37881199-37881447	249	This study
ERBB4	c.955G>A	CTTGTTTTGAGCTTGTTTGCTG	CCTTGTTATATAGGCCCAGTTCT	chr2:212578210-212578442	233	This study
ERBB4	c.1817A>G	GGGTCCTGACAACTGTACAAAG	CCAGCAAGAATGCTTACCCT	chr2:212530031-212530203	173	This study
ERBB4	c.542delA	GCAGACACCATTCATTGGC	GCATAACTCATTCATCGCCAC	chr2:212652683-212652834	152	This study
ERBB4	c.719G>A	TTCTCCTGGTGGCAGACTTT	TAGGGAAGGAAAGGAGAGCA	chr2:212589750-212589968	219	This study
FBXW7	c.1514G>A	TGCAGAGTTGTTAGCGGTTC	ATTAGTATGCCCCTGCAACG	chr4:153247176-153247388	213	This study
FBXW7	c.1393C>T,c.1394G>A	TGGGACATACAGGTGGAGTATG	CTACACAGAAAGGGCCCAAA	chr4:153249289-153249525	237	This study
FGFR1	c.352_355delGAGG, c.348delC, c.355delG	TCCGTGTTCATCTGGAACTG	CTCTTAAACCCAATGCCCAG	chr8:38285808-38286038	231	This study
FGFR2	c.1672delG	TGATGCCACAGAGAAAGACC	GGAAGCCCAGCCATTTCTAA	chr10:123257932-123258118	187	This study
FLT3	c.2011_2012insG	CATGCCTGGCTTCTCTCATA	CCACTTGGGTTTGAGAGTTC	chr13:28602174-28602562	389	This study
GNAS	c.2531G>A	TCTGAGCCCTCTTTCCAAAC	GGGGTGAATGTCAAGAAACC	chr20:57484301-57484536	236	This study
JAK2	c.1847_1851delGTGTC	TGGACAACAGTCAAACAACAA	GACACCTAGCTGTGATCCTGAA	chr9:5073651-5073877	227	This study
KDR	c.767T>C	TCTGAGTCCGTCTCATGGAA	GCAAACTTCACTGGGGCTTA	chr4:55980229-55980410	182	This study
KDR	c.4016C>T	TCACTCCGATGACACAGACA	ACTTCCTGCTGGTGGAAAGA	chr4:55946019-55946252	234	This study
KIT	c.1618G>C, c.1630delA	TGTAGAGCAAATCCATCCCC	CATACATGGGTTTCTGTGGG	chr4:55593379-55593594	216	This study
KRAS	c.59G>T	GGCCTGCTGAAAATGACTGA	GAATGGTCCTGCACCAGTAA	chr12:25398164-25398330	167	[29]
KRAS	c.183A>C	CCAGACTGTGTTTCTCCCTTCT	AACCCACCTATAATGGTGAATATC	chr12:25380161-25380371	211	This study
KRAS	c.34G>A, c.35G>T, c.35G>A	CGTCTGCAGTCAACTGGAAT	GAATGGTCCTGCACCAGTAA	chr12:25398164-25398501	338	[29], [23]
MET	c.1125C>G	ACAAATAGGAGCCAGCCTGA	GTGGGGAACTGATGTGACTT	chr7:116340131-116340360	230	This study
MET	c.3025C>T	GCCCATGATAGCCGTCTTTA	TGTCACAACCCACTGAGGTA	chr7:116411854-116412103	250	This study
MLH1	c.1151T>A,c.1153C>T	AGACTTTGCTACCAGGACTTGC	CTTTGTTGTATCCCCCTCCA	chr3:37067126-37067448	323	[29], [23]
NOTCH1	c.4717A>G, c.4723G>C	ACCAGTACTGCAAGGACCACTT	TCACGCTTGAAGACCACGTT	chr9:139399317-139399545	229	This study
NRAS	c.34G>T	GATGTGGCTCGCCAATTAAC	TCACCTCTATGGTGGGATCA	chr1:115258667-115258840	174	This study
PDGFRA	c.2515delC	CTTGCAGGGGTGATGCTATT	AAAGTGAAGGAGGATGAGCC	chr4:55151940-55152177	238	This study
PIK3CA	c.2091G>A, c.2119G>A	TCCTATTGTCGTGCATGTGG	TCTGCTTGATTCCAAGGACC	chr3:178938826-178939049	224	This study
PIK3CA	c.1031T>G	GCATTTCCACAGCTACACCA	TGGATGTTCTCCTAACCATCTG	chr3:178921447-178921696	250	This study
PIK3CA	c.3140A>G	CTCAATGATGCTTGGCTCTG	CTATGCAATCGGTCTTTGCC	chr3:178951951-178952237	287	This study
PIK3CA	c.1624G>A,c.1633G>A	CTGTGAATCCAGAGGGGAAA	CATGCTGAGATCAGCCAAA	chr3:178935934-178936201	268	[29], [23]
PTEN	c.737delC,c.752G>T	TGCAGATCCTCAGTTTGTGG	TGGATATTTCTCCCAATGAAAG	chr10:89717605-89717810	206	This study
PTPN11	c.182A>G, c.214G>A	AATCCGACGTGGAAGATGAG	CATTTCTGACACTCAGGGCAC	chr12:112888037-112888378	342	[23]
RET	c.1940delT	AGCCATGAGGCAGAGCATA	CCTCCGGAAGGTCATCTCA	chr10:43609843-43610082	240	This study
SMAD4	c.1525T>G	TGTCCCTCTGATGTCTTCCA	AAGGTTGTGGGTCTGCAATC	chr18:48604591-48604829	239	This study
SMAD4	c.1081C>T, c.1082G>A	TGGATGTTCAGGTAGGAGAGAC	CATGGGAAAACATAACCTTGA	chr18:48591829-48592058	230	This study
SMARCB1	c.1129C>T, c.1130G>A	CAGCCCAGATCCAGGTTTT	TTGCCAGAAGATGGAGGAGA	chr22:24176112-24176439	328	[29]
SMO	c.970G>A	AGTACAGAGTGACCGCCTCAA	ACAGTGAGGACAAAGGGGAGT	chr7:128845946-128846188	243	This study
TP53	c.811G>A, c.814G>A, c.818G>A, c.832C>T,	TCCTTACTGCCTCTTGCTTCTC	TGAGGCATAACTGCACCCTT	chr17:7576964-7577195	232	This study
TP53	c.376T>C, c.380C>T, c.388C>G, c.404G>A	GTTTCTTTGCTGCCGTCTTC	TCATGTGCTGTGACTGCTTG	chr17:7578422-7578637	216	[23]
TP53	c.523C>T. c.524G>A, c.535C>T, c.566C>T, c.565G>A, c.536A>G	TGTGCAGCTGTGGGTTGATT	TCCACTCGGATAAGATGCTG	chr17:7578256-7578504	249	This study
TP53	c.844C>T, c.853G>A, c.857A>T	GCCTCTTGCTTCTCTTTTCC	GCTTCTTGTCCTGCTTGCTT	chr17:7576997-7577187	191	This study
TP53	c.730G>T, c.733G>A, c.742C>T, c.743G>A	AAAAAGGCCTCCCCTGCTTG	GGTGGATGGGTAGTAGTATGGAAG	chr17:7577386-7577683	298	[29]
TP53	c.31G>C	TCAGACACTGGCATGGTGTT	ATTTTCGCTTCCCACAGGTC	chr17:7579756-7580044	289	This study
TP53	c.637C>T,c.641A>G, c.659A>G, c.660T>G	CCTCTGATTCCTCACTGATTGC	GGGAGGTCAAATAAGCAGCAG	chr17:7578070-7578317	248	[29]
TP53	c.294_298delTTCCC	AGATGAAGCTCCCAGAATGC	GCCAGGCATTGAAGTCTCAT	chr17:7579265-7579507	243	This study

Supplementary Table S7. The coverage of an individual region in 42 out of 74 gastric cancers analyzed in this study

Amplicon	Gene	Region	Length	B-GC1T	B-GC2T	B-GC3T	B-GC4T[a]	B-GC6T	B-GC7T[a]	B-GC8T	B-GC9T	B-GC11T	B-GC12T	B-GC13T	B-GC14T	B-GC15T	B-GC16T	B-GC17T	B-GC18T	B-GC19T	B-GC21T	B-GC22T	B-GC23T	B-GC25T	B-GC26T	B-GC27T	B-GC28T	B-GC30T	B-GC33T	B-GC34T	B-GC35T	B-GC37T	B-GC38T	B-GC39T	B-GC41T	B-GC43T	B-GC45T	B-GC46T	B-GC47T	B-GC50T	B-GC51T	B-GC52T	B-GC53T	B-GC55T	B-GC56T	Average
AMPL495041	*MPL*	chr1:43814962-43815050	89	1353	2433	2756	3900	1133	3328	1988	3007	2753	2448	2553	2397	2243	2803	2651	2742	3087	4939	3316	3526	4278	2969	6953	5394	5907	4147	4051	4255	2875	4906	5127	4963	4226	3580	4632	3624	3957	3913	3000	4063	3033	4919	3574
AMPL30014	*NRAS*	chr1:115256504-115256554	51	2697	3120	3088	3025	2656	3058	3227	3680	3480	2563	3048	2729	2669	3683	3007	2507	3418	2852	2877	3947	3252	1745	4445	4196	4109	2276	2566	2661	2417	3970	3844	4143	3479	2831	3445	2654	3365	2666	2945	2207	2959	3861	3128
AMPL30373	*NRAS*	chr1:115258717-115258765	49	3945	8861	9812	10521	12724	5356	4878	6480	6579	4206	4656	4907	4756	5656	6008	2724	5110	5452	5377	6828	6842	3040	8031	5667	6126	7244	5002	4655	5317	7135	5810	4915	5489	3494	4803	3138	4324	4112	3152	2681	3684	4467	5571
AMPL34014	*ALK*	chr2:29432657-29432711	55	9083	18612	17373	15722	51100	8761	8295	10449	7022	6032	7407	7162	11780	10106	6992	5287	6983	9023	11389	10218	8075	6229	10781	7728	9319	8858	9875	5823	5775	16202	6052	6299	7917	8040	5129	6113	7229	5031	5522	4144	5680	6408	9548
AMPL33013	*ALK*	chr2:29443688-29443741	54	4340	5691	5404	6496	6621	5643	3980	2622	3782	3820	3577	4264	5982	4512	3967	2211	2173	5481	4722	2942	2341	3117	6024	2158	2249	3124	3660	3129	3455	2969	4437	3922	2030	4002	4602	2826	1974	3658	2422	2371	3669	4162	3822
AMPL31381	*IDH1*	chr2:209113105-209113153	49	4443	10622	9882	10798	19636	5553	5266	6664	5977	4316	5268	4667	4826	5083	4626	3735	4622	6185	6994	6304	5995	2993	6986	4247	5008	6684	3667	4036	3833	4348	4548	4221	4508	3725	4155	3230	3575	3755	2732	2458	3559	4323	5430
AMPL31533	*ERBB4*	chr2:212288919-212288978	60	2594	3695	4552	5324	2028	5643	4374	5953	5876	4256	5077	4356	4234	5062	5092	2995	4774	5666	5711	8432	6909	2802	8102	5708	6428	6213	4693	3982	3063	4969	5627	4795	4048	4254	3353	3691	4310	4131	3178	2997	3949	5923	4734
AMPL226460	*ERBB4*	chr2:212530063-212530155	93	2836	727	1821	2209	76	5771	3981	4903	5221	4866	5179	4405	3736	4122	5075	3576	4804	10187	5533	10255	8360	3574	10105	7707	9558	7841	5491	6552	4120	9844	3554	4551	5532	6357	6724	3501	5747	5316	3478	4688	5723	6212	5370
AMPL33659	*ERBB4*	chr2:212576812-212576872	61	1852	3075	2820	2906	4013	1971	2383	3055	2711	1949	2420	2163	2399	2715	2144	2719	2357	2322	2505	2040	2180	1072	2411	1828	2310	1539	1376	1792	1457	2244	3988	2984	3446	2824	2331	2736	2521	1870	1697	2316	3105	[illegible]	2430
AMPL645344	*ERBB4*	chr2:212578292-212578350	59	1270	4705	5126	4077	4348	2520	3119	3645	3438	2783	3292	2316	2608	3031	2661	2455	2887	3757	4216	3893	4176	1561	5879	3774	5975	6457	3359	3262	2410	6831	3952	3118	4068	2887	3765	3660	3452	3194	2444	2058	2718	3792	3546
AMPL32827	*ERBB4*	chr2:212587136-212587185	50	2469	5029	4886	5138	4275	3861	4419	5873	5760	3844	4096	3954	4081	4419	3575	4117	4602	3728	4834	4337	3647	1972	5231	3678	4770	5595	3096	3854	3709	4468	4710	4092	4671	3830	3984	3944	3493	3136	2741	2252	3228	4560	4094
AMPL33781	*ERBB4*	chr2:212589768-212589840	73	3303	6047	6278	8730	2830	6071	4588	6924	6560	5124	5304	4327	4805	5203	6309	5308	5493	8941	7288	8819	8867	3628	10700	6189	7973	8391	5269	5199	4432	6259	6211	5364	5962	5123	6061	4236	4811	5584	3047	3576	4796	6643	5871
AMPL32781	*ERBB4*	chr2:212652717-212652775	59	473	486	310	252	189	442	671	696	578	709	671	604	827	819	1423	736	836	674	1289	796	817	516	1046	949	1526	555	673	420	339	3059	2714	1752	2415	2442	2253	1781	2283	709	1089	1570	1099	1708	1076
AMPL33555	*ERBB4*	chr2:212812107-212812169	63	223	250	166	164	158	255	423	423	408	428	411	336	498	552	620	343	439	520	738	369	509	284	551	548	776	289	342	187	161	1160	2184	1242	2032	1700	2010	1906	2031	731	950	1520	1014	1491	746
AMPL613072	*VHL*	chr3:10183795-10183859	65	1073	4181	4573	4303	5026	2119	1857	2610	1874	2013	2337	2086	1707	2166	1971	1809	2032	3490	2312	2621	3133	1796	3939	3791	5114	3005	2897	3333	3127	3928	4047	5743	4102	3603	4271	2737	3402	3172	2868	2912	3411	3671	3099
AMPL232121	*VHL*	chr3:10188216-10188302	87	8756	13026	13011	12823	11356	6254	6781	8413	7289	6896	8799	7573	6832	7356	7225	3977	7217	10540	7787	10278	7673	6234	8160	8046	10434	5379	6200	2837	5855	9983	6012	9236	7463	6366	7524	6424	7339	6322	4735	5871	6830	7679	7733
AMPL34860	*VHL*	chr3:10191478-10191532	55	8867	12290	12981	11553	16532	6625	6722	8085	6790	5954	7972	6181	6217	6573	6496	4286	5591	8106	7477	9241	6801	6109	6730	7357	9368	4735	4808	5264	4936	7398	6782	9568	6302	4831	6587	5089	5749	5217	5669	4757	5914	6304	7162
AMPL38772	*MLH1*	chr3:37067208-37067266	59	5867	11518	11159	10737	5932	7474	8695	10708	7921	6206	8349	8188	6936	8385	7826	4107	8600	12251	10095	12296	8476	7657	12719	9527	14931	7747	6695	6407	6732	23194	8174	10607	8389	8424	8615	8132	9469	7172	7889	6439	6124	8497	8935
AMPL423052	*CTNNB1*	chr3:41266039-41266137	99	5274	7186	5979	8940	3459	6269	5051	6340	5465	4478	5666	5785	5188	5811	5412	3778	5769	8413	5755	9300	7960	5347	8473	6564	7911	3897	4117	5047	3877	7054	5435	7616	5383	4811	6235	4260	4921	5408	4099	4556	4229	6734	5792
AMPL36101	*PIK3CA*	chr3:178916844-178916907	64	192	453	548	618	225	1739	1158	1727	1656	1647	1356	1637	1069	1312	1667	1522	1805	2376	1966	3213	2278	1945	4554	2869	2903	3085	1377	1575	1352	40197	2198	2364	2907	1864	1566	2496	2578	1590	1453	1664	1981	2062	2732
AMPL554194	*PIK3CA*	chr3:178921502-178921570	69	505	1539	1073	586	1279	485	798	997	898	687	980	1429	1185	1260	628	1259	898	389	826	313	363	552	709	584	841	416	440	630	636	24707	2155	2138	3262	1673	2599	2495	2209	1684	1424	2104	1973	1877	1750
AMPL36569	*PIK3CA*	chr3:178927931-178927986	56	211	179	113	131	59	405	444	441	442	572	409	601	436	660	867	546	713	673	1153	487	728	886	1063	863	1176	383	339	295	285	20665	2066	1753	1723	1420	1522	1126	1057	478	612	658	601	813	1192
AMPL35831	*PIK3CA*	chr3:178936057-178936111	55	1950	5407	3357	2560	4358	2489	3866	4436	3525	3376	3679	5151	3592	4363	2717	5144	3597	1829	3486	2101	2053	2408	3840	2551	3295	1894	1310	2385	1772	85597	4107	4937	6016	3747	4594	4817	3261	3274	3851	3740	3968	3736	5432
AMPL339432	*PIK3CA*	chr3:178938822-178938906	85	1651	3022	3531	2729	2362	2327	2101	2258	1869	1900	2664	2565	1818	2364	1239	1751	1663	4904	4862	3802	4373	2553	3924	2565	4330	2576	1660	1636	1367	58708	3139	3540	3425	2283	2350	2009	1724	1311	930	925	1302	1456	3797
AMPL118631	*PIK3CA*	chr3:178952002-178952091	90	3419	20720	14835	14246	29093	2846	2614	2767	2036	1661	1682	2753	2132	3270	1559	3099	2371	1514	4392	2035	1964	2091	3868	1976	3276	3853	2301	2432	3671	43118	2147	2807	3906	2089	2579	2155	2246	1662	1736	1868	1635	2032	5106
AMPL37385	*PIK3CA*	chr3:178952139-178952190	52	1753	2460	2205	1523	1491	2673	2145	3272	2777	2796	2559	4542	2798	2981	2520	2754	3151	2662	2487	3391	2858	3840	5698	4117	2894	1528	879	2067	1731	35439	5022	5626	5903	3672	5617	3492	4581	4541	2329	3668	4548	4500	3988
AMPL508777	*FGFR3*	chr4:1803549-1803626	78	1961	4758	4858	5310	7704	1255	1627	1732	1254	1268	1243	1320	1536	1831	1242	1617	3890	1434	1742	988	1374	997	2576	2136	3711	2220	2020	2268	2382	4077	3268	3358	3177	1999	2361	1984	2293	2364	2120	1057	1775	2470	2394
AMPL142574	*FGFR3*	chr4:1806111-1806208	98	12039	7236	8303	13250	3044	7028	5735	6659	5817	4624	5962	5192	5197	4891	5288	4146	14693	9210	5497	11069	7695	6812	8799	6288	6817	3452	3638	5458	4043	6238	6245	5588	5367	3948	6600	4020	4753	7318	3661	2704	4886	6672	6331
AMPL411633	*FGFR3*	chr4:1807841-1807931	91	4983	4830	5114	6274	3523	3530	3663	3922	3284	2699	3088	3031	3054	3179	3274	2827	8912	4835	3467	2579	4457	3738	4498	3693	6333	2578	3275	3282	2724	7172	3629	3170	3718	2602	3204	3472	3229	4104	2908	1725	3215	3889	3826
AMPL493041	*FGFR3*	chr4:1808283-1808378	96	14844	17192	13993	17411	21124	7865	7948	8349	6196	6209	7487	6407	6929	8109	6617	2966	19327	11199	8375	11456	8631	8010	9143	7352	10306	5089	5304	6311	4504	10452	6359	6746	6791	5358	6396	5252	6321	7558	5279	3617	6215	7938	8546
AMPL505293	*FGFR3*	chr4:1808938-1809007	70	5613	4107	5891	8050	2225	4711	3918	5359	4026	3579	4151	3577	3939	3812	4458	2885	11610	7942	4145	7241	6243	5440	6144	4977	5138	2953	2832	4080	3634	5346	4947	4327	4445	3109	4246	2683	3820	4745	2812	1957	3856	4767	4613
AMPL43181	*PDGFRA*	chr4:55140988-55141063	76	6096	9629	11149	11617	7586	6564	7021	8734	6349	6646	6729	5377	5497	6370	6134	5125	6080	9510	6800	10330	9313	16808	8210	7251	10333	5546	4567	6097	5373	8186	6853	6825	7098	5675	6582	5000	6020	4918	5446	5201	5981	7061	7231
AMPL43383	*PDGFRA*	chr4:55144112-55144180	69	6425	19842	15858	15826	13479	6496	6952	7451	6899	6483	6695	5924	6316	7034	5378	4026	5414	7375	7768	8160	7537	14391	5895	5072	6619	3906	4270	4798	4588	6438	5308	4460	5223	3669	5055	3906	4145	3445	3186	3876	3758	4530	6759
AMPL43200	*PDGFRA*	chr4:55144521-55144588	68	5564	12443	9511	8335	8453	4311	6955	7958	6422	5241	6582	5395	6180	6684	4876	4643	5195	3905	4932	4617	4388	10379	6142	4606	6652	3582	3273	4975	3829	6515	5968	5427	6991	5147	6441	6846	6355	5059	5805	5356	5370	7679	6071
AMPL234145	*PDGFRA*	chr4:55152023-55152109	87	15965	53087	43526	36927	89579	11917	16037	16597	13340	13894	15001	13473	13606	14718	12965	11597	13241	15684	14076	17654	14317	30427	14582	12923	17752	11652	10281	11926	11508	24149	10956	10972	12769	9213	12141	10241	11935	9251	9794	11073	9814	13306	17711
AMPL44733	*KIT*	chr4:55561749-55561816	68	3407	10207	12318	9013	7895	4888	6027	6950	5264	5434	6094	4727	4839	5616	4357	2450	5001	6653	6115	7067	6421	11174	6505	5800	8448	5153	4069	5011	4519	9125	5501	5366	6409	4104	4697	4917	5678	3887	4388	4126	4038	5257	5927
AMPL46436	*KIT*	chr4:55592147-55592230	84	331	349	195	178	150	274	489	453	350	576	450	673	600	612	998	353	644	484	743	551	564	1624	686	720	1040	224	378	329	320	1629	2210	1314	1532	1162	1442	843	892	351	703	729	533	827	703
AMPL46166	*KIT*	chr4:55593449-55593509	61	939	1083	899	767	452	730	1121	1622	2021	1169	1870	1268	1745	1416	1562	927	1466	1287	1020	1204	1082	2885	1288	1283	1362	273	491	852	557	1032	3510	2223	3102	2327	3518	2141	2666	1839	1355	2318	1919	2866	1559
AMPL178642	*KIT*	chr4:55593582-55593689	108	2720	9060	6475	6689	3399	1096	1489	1804	1387	872	920	756	955	1007	719	415	814	936	1083	917	681	1467	814	624	995	788	937	851	629	1473	493	613	1067	892	802	805	884	537	636	672	553	631	1461
AMPL47704	*KIT*	chr4:55594220-55594294	75	5170	10901	8300	9444	7307	6231	6119	7849	7575	5611	6058	4992	5220	5566	6173	3073	6360	10254	7114	10614	8693	16233	9085	8057	11193	5242	4844	6022	4393	11131	5785	6384	6337	5241	5751	5882	6385	5316	5580	5267	5686	8084	7060
AMPL48478	*KIT*	chr4:55595496-55595562	67	1479	3823	3220	2314	2684	1417	2988	3529	3231	2562	3284	2753	3519	3346	2266	1630	2677	1255	1388	1497	1550	4124	2967	2564	3095	1021	1337	2315	1363	3741	6514	4257	5737	3629	5118	5409	5747	3349	3925	4497	3927	4965	3143
AMPL47725	*KIT*	chr4:55597468-55597535	68	6134	8341	10064	11628	3892	7777	7327	9326	7613	7177	7040	6940	6519	6946	6913	5673	7301	9877	6982	11599	9209	17396	9705	8200	9236	5038	5291	7233	5751	5751	8318	7137	6276	5128	7390	4941	6399	5428	4204	5582	5374	8578	7444
AMPL49160	*KIT*	chr4:55599295-55599365	71	1821	2025	2464	2453	1215	2752	2712	3267	2486	3158	3230	2650	2582	2972	2728	1841	3213	2686	2147	3658	3229	6377	4609	4471	4304	1459	1934	3275	2793	2931	3661	3277	3677	3161	3468	2940	3787	3236	2841	3426	3597	5005	3084
AMPL49191	*KIT*	chr4:55602686-55602740	55	4221	7734	6562	7416	8715	4430	4522	6128	4562	4317	4117	4009	4000	4306	4498	2763	4240	6788	4153	6733	5873	11578	5093	5004	5690	2794	3171	4204	4612	4274	4975	4752	5410	3986	4668	3268	4076	2990	2998	3378	3383	5095	4892
AMPL49305	*KDR*	chr4:55946111-55946171	61	3276	3894	5489	6497	2394	5715	3403	4990	4331	3858	4063	3454	3191	3770	4615	1984	3865	6937	3702	8209	7031	11244	6274	4890	4980	2739	2554	4027	3179	3078	4787	4903	3848	2659	4589	2391	3183	3158	2265	3367	3796	4719	4317
AMPL49402	*KDR*	chr4:55946218-55946277	60	7326	17267	18088	16444	26593	6950	8690	10419	7525	7882	7794	7935	7264	8406	7489	6123	7303	10211	8881	13280	9994	18430	9830	8347	11281	7023	5279	7253	6538	11171	7304	7654	7875	5974	7016	5971	6862	5162	6134	5579	5546	7340	9177
AMPL50673	*KDR*	chr4:55953790-55953860	71	4893	7616	8096	9739	3531	5791	6309	6662	6134	5930	5845	5144	5448	5964	5827	4443	5776	9651	6491	8897	8166	13826	8375	7654	10102	4241	4504	5267	3708	6757	7433	7434	6764	5310	7109	5663	6035	4853	5720	4701	4864	7610	6530
AMPL50695	*KDR*	chr4:55955062-55955132	71	3713	9288	9793	9707	5807	4714	5191	6575	5430	5417	5240	4905	5219	5049	4702	4324	5207	6070	5610	6958	6211	12024	6928	5788	7660	5107	3949	4776	3357	6271	6523	5197	5726	4283	6008	4140	4855	3831	4006	3815	4098	5627	5693
AMPL52170	*KDR*	chr4:55960987-55961064	78	7371	23353	19698	16378	16800	8187	10775	10348	9276	8166	9048	7211	7175	8390	7332	4370	8112	10732	10765	12578	10786	22617	11143	9028	13115	7040	5927	6935	5855	19311	8288	7731	8548	6688	8175	7715	8471	5650	7175	6897	6486	8510	9956
AMPL52189	*KDR*	chr4:55962450-55962522	73	4680	9881	9322	9123	9058	5751	6612	7238	6838	6093	6354	5147	5198	6103	5472	4043	5860	9427	6072	8714	7692	14484	7424	7037	10963	4989	4373	5293	4611	10270	4091	5236	5843	5370	4955	6536	6375	4277	5090	4703	5078	5790	6606
AMPL52386	*KDR*	chr4:55972921-55972974	54	1616	2969	3797	3895	2087	3150	3613	3636	3016	3494	3304	2848	2827	3077	3669	2736	3682	5691	3993	5353	4824	7919	5742	5239	6327	3543	2999	3620	2364	7841	4693	4041	4114	3351	3452	3989	3699	3089	3801	3217	3563	5685	3942
AMPL52394	*KDR*	chr4:55979608-55979656	49	2556	7021	5141	4672	6460	2702	3733	5092	4486	3465	4371	3604	4686	3935	3306	2255	3207	1996	2506	2536	2199	6246	3165	3093	3618	1625	1929	3056	2071	2885	6595	5154	6206	4134	4954	4869	5984	3472	4052	4905	4114	4473	3965
AMPL52210	*KDR*	chr4:55980318-55980372	55	499	456	626	490	77	681	983	1418	1090	1080	1212	1158	1273	1179	1042	268	1019	666	624	719	790	1752	1110	1087	1220	330	539	828	386	1438	3304	2762	3067	2529	2983	2717	3155	1786	1726	2546	2342	2862	1377
AMPL40184	*FBXW7*	chr4:153245442-153245493	52	3237	5868	8150	6690	2561	5652	5076	6202	4619	3908	5776	3738	4370	5078	4051	2311	4903	5925	5394	5152	6218	3326	6482	4967	5885	4805	3277	4074	4720	6253	5662	3625	5305	4091	4629	4346	4445	2898	3250	3485	3836	5255	4750
AMPL40234	*FBXW7*	chr4:153247285-153247369	85	8030	8429	8369	13350	5502	9009	6538	8784	8049	6546	7369	5857	6379	6951	7152	4743	8240	9911	6582	12512	9532	3101	11104	7426	8421	5108	4212	6328	5307	5727	6924	7487	6514	5065	8100	4390	5237	4506	4194	5765	6134	9608	7107
AMPL41691	*FBXW7*	chr4:153249363-153249429	67	4295	3785	4842	5111	1097	5500	5553	6740	5714	4469	5710	4392	4970	5165	4833	2376	5264	6283	4349	6839	6133	4255	7510	6415	6888	3564	3571	4230	3941	5340	7294	6942	6283	4472	6237	4774	5489	3082	4148	4105	4404	6322	5064
AMPL41812	*FBXW7*	chr4:153250860-153250918	59	4110	9223	7942	6825	8937	3502	4890	5533	4415	2978	4615	3552	4539	4561	3357	1838	4156	2357	4063	2566	2961	2287	3116	2494	3780	2176	2248	3099	3244	4013	4797	3823	4464	3523	3860	4445	4418	2500	3380	3300	3344	4177	4034
AMPL42297	*FBXW7*	chr4:153258979-153259025	47	232	255	370	328	106	576	598	943	781	604	834	579	843	683	706	418	812	642	386	682	655	452	767	823	759	319	363	707	477	587	1987	1650	1842	1353	1365	1377	2113	984	796	1290	1259	1669	833
AMPL53695	*APC*	chr5:112173884-112173947	64	4006	13973	14219	12298	14411	5233	6989	7753	5974	4696	5902	4950	7789	5737	4983	4856	5822	8272	7383	8367	7669	4714	8895	6230	10208	4719	6615	5334	4494	9876	6119	6017	7260	5101	6352	5796	5966	3675	5749	2936	5786	5883	6881
AMPL54189	*APC*	chr5:112174605-112174655	51	2107	2508	2942	2873	1792	2725	2953	4634	3920	2512	3433	3139	4620	2876	3158	1920	3365	3096	2678	3717	3203	2004	4466	3132	3944	1489	2069	2848	2057	2751	4493	3445	3972	3326	3313	3860	3681	3144	2864	1786	3846	4144	3114
AMPL320120	*APC*	chr5:112175158-112175255	98	4370	7624	7677	9907	7879	5973	4159	6081	5745	4437	5302	4679	5888	4439	5366	4360	5682	10195	4284	9359	7939	5049	7964	6747	6756	3810	4937	5202	5134	5063	5376	4867	5417	3926	6170	4097	4785	4503	2711	2802	5337	6183	5671
AMPL227540	*APC*	chr5:112175337-112175435	99	2745	2559	3903	6473	684	6147	3343	5057	4762	3179	4526	3360	5270	3328	4985	3120	4621	8742	3961	7897	7232	4354	6921	5005	4674	2772	3920	3712	3485	2777	3941	3311	3924	2667	3490	2591	3373	3860	1610	2008	4221	5249	4137
AMPL145156	*APC*	chr5:112175581-112175690	110	2412	4583	4495	5530	3975	2408	2027	2813	2888	2447	2674	2398	3719	2574	2612	1583	2735	4578	3257	3968	3508	2332	3975	3654	4644	2435	3538	2755	1802	3115	2308	2351	2982	2612	2557	2639	2933	2113	2091	1682	3010	3058	2994
AMPL59934	*APC*	chr5:112175752-112175816	65	3344	8314	7447	8229	4644	4476	5465	7271	6443	4201	5755	5581	7165	5565	5223	5095	6024	5766	6452	6640	5772	4501	8013	5491	7668	4364	5151	4789	4459	5915	6088	4564	6266	4155	6384	5053	5128	3897	4740	2557	4614	5716	5581
AMPL60049	*APC*	chr5:112175938-112175982	45	62	50	79	58	30	110	109	141	102	158	163	174	245	153	239	101	183	207	181	178	209	179	243	241	281	97	141	113	84	383	733	464	604	412	470	407	634	197	195	260	388	465	236
AMPL60239	*CSF1R*	chr5:149433629-149433695	67	4373	8810	9834	9340	7724	5587	5176	6077	6110	4414	5252	5499	6897	5559	4970	5134	5441	7342	8192	7557	6932	4570	8347	8022	9349	5256	6962	6461	4774	8016	6893	7020	6559	5181	5824	5245	5937	5795	5592	4233	6486	7072	6424
AMPL59674	*CSF1R*	chr5:149452992-149453051	60	3291	5142	7496	7708	2847	6119	4395	4844	5361	4133	4200	4073	5454	4145	4777	2354	4603	7857	5333	7790	5246	4381	6755	5115	5230	2914	4887	4555	3408	3812	4645	4173	4052	3051	3745	2878	2974	4162	3209	2960	4176	4839	4597
AMPL236158	*NPM1*	chr5:170837502-170837599	98	151	19	107	86	9	626	525	927	861	676	1048	753	881	551	954	400	897	1062	364	961	1250	422	2353	1569	1130	497	970	888	519	507	899	869	1437	874	1306	1356	1594	1387	379	1139	1864	1705	875
AMPL307725	*EGFR*	chr7:55211024-55211132	109	10336	18216	14870	14156	14382	9266	8594	9419	9767	6430	8982	7044	10603	10027	7017	8937	8303	10125	12110	11017	10917	9198	12092	8229	12689	9239	10839	6335	4768	13435	4940	8856	8441	6110	7493	7871	7498	8629	6335	5254	10156	8238	9456
AMPL68178	*EGFR*	chr7:55221796-55221866	71	6881	6755	8113	10573	6953	8815	4047	6031	7494	4585	4782	4985	6253	5127	5902	3594	5230	10281	6109	9495	7333	7357	8492	5582	5686	6379	4700	4606	4577	5644	6157	8147	5220	2927	5651	2461	4498	5684	2585	3181	6484	5437	5971
AMPL1594224	*EGFR*	chr7:55232962-55233053	92	13487	42294	32317	28165	54621	4831	13314	13178	13861	5565	11534	10541	16568	13964	9311	5837	9710	13803	18107	14820	12247	12740	14794	10809	15753	13919	13267	9064	8015	28756	8461	13517	10663	7520	9182	10951	10203	11170	9950	7151	12452	9213	14420
AMPL67641	*EGFR*	chr7:55241644-55241717	74	670	281	793	1034	45	0	1749	2573	2687	2336	2075	1939	2141	1813	2238	1665	2058	6130	2350	5386	4511	4165	7553	5589	5552	4053	4076	3803	2999	4608	4001	5891	4413	2366	4455	2809	3912	4345	2506	2791	5944	4168	3202
AMPL1598122	*EGFR*	chr7:55242416-55242540	125	2242	952	1796	2313	94	0	3201	3553	4936	3884	4386	3650	4542	3735	4040	2829	3896	8470	4897	8493	7893	6695	9216	6726	7688	5294	6049	3948	1735	6445	3168	4863	4785	3620	5288	4168	4642	6559	2652	3534	7810	5132	4520
AMPL493236	*EGFR*	chr7:55248999-55249072	74	2504	3219	3530	4580	3123	0	2010	2416	3088	1717	2114	1885	2624	2427	2676	1825	2457	4631	2610	4684	3834	4101	4207	2672	2389	1928	1780	2111	2069	1783	2618	2641	2149	1179	2843	1029	1377	3108	1193	1266	2958	2758	2527
AMPL174184	*EGFR*	chr7:55249109-55249187	79	9534	30984	24455	24824	44381	0	8850	8749	11064	6560	7896	7686	11960	10310	7364	9200	8551	9924	12669	9437	8953	8599	13069	9260	13330	11892	10620	7648	6996	19970	7648	10563	9850	6405	8564	8207	8049	10025	7176	5755	11202	9817	11381
AMPL1599089	*EGFR*	chr7:55259432-55259539	108	17188	33852	23499	25918	42900	0	13287	12488	15219	10215	12539	9901	14786	14276	10111	5807	12179	9492	17046	18284	14403	15626	16105	12585	17752	13157	12963	8274	6038	24199	8999	14819	11511	8232	10485	9562	11709	11385	9366	8324	13734	11667	14045
AMPL61096	*MET*	chr7:116339618-116339697	80	4849	5568	6270	6768	3638	0	9143	6386	6080	4445	5278	3852	6156	4788	5353	4918	4322	11204	7888	9427	8714	5121	8960	7972	8594	4978	6005	4843	4094	5748	6286	9344	6330	4722	6360	3699	5271	4983	3937	4849	4958	6415	5917
AMPL61118	*MET*	chr7:116340222-116340275	54	5222	7634	6251	6796	10621	0	8146	6430	5967	3860	5423	3861	7437	6148	4674	4387	4845	4779	6494	4652	4201	3244	7013	4790	4771	2688	3300	4761	4141	3955	5471	6915	7049	4325	6153	4646	5030	4701	3524	4419	4028	5938	5207
AMPL62593	*MET*	chr7:116411935-116412010	76	3159	3987	4719	4814	1560	0	8337	5707	5068	4466	4883	3722	5429	5244	4447	4731	3592	9007	8054	7445	7143	3960	8565	6542	7978	5744	6633	4751	3964	5619	6181	7965	6674	4348	6193	5465	5316	4058	4094	4450	4168	5914	5336
AMPL62615	*MET*	chr7:116417428-116417506	79	5169	16710	9936	10622	16969	0	11335	7938	6641	4450	5837	3741	6748	6536	4414	4136	5187	8291	11572	7285	9537	4233	8828	6778	9210	6041	7620	5465	4318	9592	4440	6674	7589	4688	5315	5919	5667	3684	5297	5305	4297	5864	6902
AMPL64212	*MET*	chr7:116423412-116423475	64	6173	13915	13269	12252	12627	0	14724	8947	8354	5733	7923	5710	9053	8663	6024	7578	6821	10598	13361	8288	7642	5307	13787	9899	13514	10242	11416	8421	7720	9633	7885	11184	11312	7755	8717	9351	9783	6035	8433	7388	6136	8903	9059
AMPL133212	*SMO*	chr7:128845064-128845159	96	8138	22265	14863	15548	12674	0	8235	9516	8912	7518	9101	6398	10314	10364	7692	8882	8043	11460	15715	11314	9504	8710	12654	10274	16003	9962	11095	7949	6782	23468	7444	10510	11495	7771	9049	9136	10236	6298	8413	7655	6378	9706	10177
AMPL122008	*SMO*	chr7:128845999-128846088	90	8406	13628	12329	17997	12977	0	5491	9118	8043	6665	6921	5317	8513	7416	7533	8782	7127	12198	10215	10535	10497	8058	11910	7536	7732	6610	7859	2302	7081	8438	6302	9498	8137	4350	7481	4586	5769	5541	3604	5791	5080	9607	7928
AMPL64101	*SMO*	chr7:128846361-128846416	56	125	75	123	252	21	0	439	685	793	728	576	462	619	500	1054	259	815	2620	764	1844	1800	1063	4819	3547	2575	2135	1858	1995	1380	2346	3033	3484	2369	1583	2144	1182	1954	1968	1200	1854	2077	2797	1474
AMPL482180	*SMO*	chr7:128850285-128850363	79	5107	6807	6089	9015	10154	0	3025	4231	4431	3231	3454	2570	4440	3697	3966	2536	3527	7564	5427	5563	5237	4888	5656	4003	3447	2845	3224	3139	3738	3077	3669	4287	3154	2123	3308	1782	2420	2495	1951	2473	2284	4359	4009
AMPL492615	*SMO*	chr7:128851552-128851614	63	6061	9795	8587	9470	11272	0	4517	6359	5040	4043	4655	3233	5778	5208	4585	3746	4915	8788	8971	7454	6403	5306	5787	4236	4413	3537	3672	3302	3007	4501	4072	5473	4212	2832	4042	2751	3080	3086	2494	2608	2563	3938	4947
AMPL304699	*BRAF*	chr7:140453120-140453223	104	5274	10109	8808	9662	8724	0	5537	6462	6704	5124	6563	4100	7364	6587	4467	5758	3577	7185	9293	9772	7829	5449	9899	5594	7045	6324	7797	6253	4279	6767	1983	3809	5222	3477	3964	5433	5513	4044	3747	5537	5174	6121	6008
AMPL1308445	*BRAF*	chr7:140481395-140481504	110	9935	29508	24386	20531	43053	0	10045	9886	8715	8075	10052	6246	12583	11309	7451	7325	7767	13061	18229	10466	10546	8447	12113	9307	13737	10330	11214	6866	5229	27877	5232	7913	9914	7037	7331	9299	7253	5374	6469	6916	5282	7701	11191
AMPL71577	*FGFR1*	chr8:38282155-38282216	62	3674	9125	9295	11816	9889	0	5137	8708	5768	5947	3954	4466	4653	5688	6190	2778	5842	7237	6174	6492	7118	4806	11173	6077	7338	7621	6480	5006	4743	8655	7482	8477	5907	5001	5605	4756	8230	7832	5379	2748	4074	7398	6303
AMPL71600	*FGFR1*	chr8:38285895-38285951	57	6104	13530	12499	10916	5327	0	9217	12243	7694	6786	6773	6127	6729	9450	9281	6582	9037	9287	10013	9757	8925	7895	13732	8441	13141	9795	11366	7178	7687	26983	8735	10528	9120	8718	10558	9125	12157	9607	8431	4389	5132	8791	9233
AMPL74598	*JAK2*	chr9:5073732-5073791	60	1325	4956	3800	4615	3443	0	4157	6281	4772	3521	5492	4926	6352	4547	4007	3662	4424	3575	7686	3494	3457	2164	5885	3988	4202	1370	1580	3847	3843	2123	5583	3944	4908	2007	4835	4065	3753	3935	2830	2139	10013	6247	4089
AMPL483802	*CDKN2A*	chr9:21971131-21971219	89	8260	23779	15156	17054	18674	0	5937	7076	6630	5240	7843	4788	8334	7126	5742	5746	6116	9056	13996	8835	7263	4182	4185	5911	7432	2921	2721	4610	5744	5276	5431	4946	4337	2287	5492	4841	4692	3986	3960	2652	7642	5984	6950
AMPL73150	*ABL1*	chr9:133738311-133738379	69	5722	15275	12296	15498	24663	0	6571	8343	6594	6010	6116	5868	7468	6443	5143	6657	5541	8457	10291	7900	7073	5477	8523	5756	7757	10889	6647	4800	6410	8769	6025	8867	7807	8207	5635	4758	5931	4974	3688	4389	3662	6499	7462
AMPL73075	*ABL1*	chr9:133747483-133747542	60	5053	10616	7058	8437	10554	0	7016	7016	5967	5449	6203	5563	7345	6728	4649	3682	5603	5756	9350	7445	6283	4351	6752	5834	7725	6673	5796	4023	4533	10216	4343	6911	5547	7467	4492	4943	6042	3602	4592	4639	3785	5514	6037
AMPL73104	*ABL1*	chr9:133748241-133748296	56	4465	9437	9187	10735	10256	0	4794	6415	4519	4559	4975	4653	6215	4645	5243	4053	5620	7799	7121	8081	7009	4735	6724	5713	6927	6642	5015	5309	6836	8007	6696	8360	5899	5964	5905	3191	3912	4493	3421	4114	3983	5947	5895
AMPL491413	*ABL1*	chr9:133748387-133748452	66	6648	16439	13810	16630	24868	0	7101	8411	6431	5982	6512	6369	8989	6987	6029	5965	6449	7106	9524	8506	7403	5228	6556	5914	7787	8510	5850	5512	5913	6825	5568	7773	6160	6442	4998	4218	4830	3947	3813	4145	4033	5822	7286
AMPL74743	*ABL1*	chr9:133750330-133750405	76	4268	7758	7377	9709	10125	0	3483	4704	4199	3738	3628	3456	5032	3751	4281	2710	3842	6651	5606	6739	5131	3932	4888	4134	4309	4423	3559	3319	4460	3438	3875	5041	3932	4115	3688	2107	2858	3004	2012	2666	2583	3848	4342
AMPL482609	*NOTCH1*	chr9:139397712-139397791	80	2123	2659	8082	3354	9397	0	4758	4273	4417	3205	3568	3318	3987	3128	4447	3579	3838	8707	6407	5960	5903	3529	8137	7928	8051	9314	9272	5547	7501	8433	7336	7849	7787	7416	6507	4563	6400	5300	5483	5243	4858	6492	5668
AMPL341576	*NOTCH1*	chr9:139399330-139399446	117	1089	453	1070	1902	46	0	2181	3048	3051	2687	2839	2561	2916	1978	2693	760	2919	4796	3112	4079	4488	2500	6266	5042	5225	7293	6443	4184	4209	4841	4548	5926	4866	4568	4881	3202	4447	4206	2761	3761	3489	5142	3487
AMPL559669	*RET*	chr10:43609070-43609162	93	3458	5723	4882	5440	4031	0	1374	2120	2095	947	1322	1127	1118	1199	1145	1233	1291	1959	2181	1545	1339	615	983	1122	936	891	1132	1190	981	1663	1114	1220	1559	1100	1428	829	836	833	711	527	1250	1202	1611
AMPL492612	*RET*	chr10:43609936-43610008	73	5195	4553	5990	0	3913	0	3637	5803	5017	4007	4432	3840	3793	4165	5026	3036	5610	7979	6144	8000	6598	3347	5701	4953	4527	3379	3356	4577	4889	6157	4772	5046	5323	3646	5068	2271	3791	4216	2331	2145	5226	5124	4442
AMPL78961	*RET*	chr10:43613796-43613918	123	182	365	469	0	198	0	387	549	450	346	321	802	356	384	442	366	534	570	617	430	486	212	1122	967	1234	936	991	1031	1132	2073	1231	1285	1661	958	1188	840	904	868	774	510	1066	1012	720
AMPL78994	*RET*	chr10:43615561-43615622	62	2084	4137	6622	0	1818	0	4851	5938	6205	3045	5007	3568	3351	4348	4141	3024	5649	6136	6965	6830	6557	3104	6822	5308	7689	4702	5137	5467	3859	12531	6676	5510	8091	5425	5704	4901	4877	5063	4660	2122	5712	5811	4987
AMPL79119	*RET*	chr10:43617404-43617456	53	2353	2758	2834	0	1689	0	2961	4568	3546	2274	3280	3397	3238	3144	3063	2198	3467	2742	4203	3136	2840	1511	3934	3282	4284	1422	1982	2576	1801	6812	7148	5791	10260	7552	7701	7213	6853	5793	5061	3354	6996	5065	3907
AMPL81777	*PTEN*	chr10:89624241-89624298	58	372	490	555	0	612	0	517	570	471	363	552	399	392	450	298	381	341	342	541	472	496	318	248	366	480	219	251	343	275	842	409	348	443	239	382	303	317	282	279	151	377	288	376
AMPL464386	*PTEN*	chr10:89685262-89685356	95	108	83	103	0	13	0	256	283	377	227	312	313	448	391	335	63	336	260	402	303	324	278	259	532	542	147	308	187	121	1523	1405	993	2268	921	1978	2735	2402	846	762	1353	1750	1325	657
AMPL80635	*PTEN*	chr10:89711882-89711932	51	420	321	315	0	122	0	626	774	638	543	692	573	737	787	746	475	886	828	1182	702	761	728	702	1042	1680	318	736	516	475	1967	3114	1935	2680	1229	2593	1698	2516	933	1156	1310	1949	1984	1033
AMPL82152	*PTEN*	chr10:89717581-89717639	59	1670	2254	4045	0	993	0	3906	4880	4246	3097	4653	3382	3633	3624	2047	2770	3429	3245	3872	3441	3043	2350	3332	4316	4699	2549	2891	3128	2171	4682	5216	4574	5139	2234	4208	4834	4619	3816	3931	2376	5410	5592	3436
AMPL421278	*PTEN*	chr10:89717694-89717773	80	6116	17226	15936	0	18898	0	8079	9495	9856	6382	8589	6415	7367	9085	5116	2623	8090	8240	11688	9700	8004	6590	5634	7783	11166	6192	7739	6431	5169	11067	7322	6124	8064	3146	8072	6825	6970	5474	6441	3378	8062	7253	7662
AMPL484297	*PTEN*	chr10:89720695-89720747	53	1943	3423	2579	0	2765	0	1930	3068	3506	1809	2011	1952	2737	2515	1360	1419	1983	1300	1979	1311	1420	1548	1262	1944	1929	638	946	1129	877	3413	4832	2478	4141	1431	3885	2620	3283	2130	2417	1963	3751	2929	2156
AMPL416888	*PTEN*	chr10:89720795-89720884	90	1075	1516	828	0	717	0	1465	1760	2391	1083	1745	1566	2166	1731	1095	1419	1910	711	988	853	738	737	948	1409	1441	298	687	1147	496	2731	1955	1951	2997	1426	2898	3050	3480	2418	2005	1601	3371	2875	1564
AMPL76043	*FGFR2*	chr10:123257996-123258043	48	1988	1850	2549	0	905	0	2715	3442	3688	2747	3333	2896	2971	2968	2472	1476	3097	2724	2615	2423	2463	1947	5156	3127	3441	1696	2609	2876	2649	3532	5360	3967	4193	3845	4673	3064	3737	4599	2431	2448	4728	4883	2959
AMPL76049	*FGFR2*	chr10:123274734-123274819	86	1867	917	1644	0	144	0	4122	4968	5578	4063	4450	3911	3538	3398	4582	3168	4762	8671	5431	8703	7442	4580	10557	7972	10674	4106	5924	4877	3672	16069	6338	6632	6618	6024	6810	6035	6979	7043	5609	4107	8046	7413	5415
AMPL77536	*FGFR2*	chr10:123279481-123279543	63	6667	6625	7926	0	2670	0	7241	8772	8398	6307	7016	5852	5829	6868	6571	2130	7178	8811	10991	10603	8158	6893	11494	8905	11831	5560	6933	5568	4643	22139	7272	7966	7688	7864	7116	6627	7718	7251	6943	4678	8585	6832	7265
AMPL136501	*FGFR2*	chr10:123279630-123279717	88	6243	11774	12570	0	11448	0	6807	9012	8190	7038	6838	6654	6688	7214	5673	4812	6944	7616	11169	9765	9433	5557	12347	7451	9340	4975	6965	5244	2950	13196	7469	8484	7674	6372	7055	6632	6784	8012	4836	4119	7232	7307	7283
AMPL120430	*HRAS*	chr11:533820-533912	93	5687	8228	5586	0	4893	0	5830	7059	7477	5765	6890	6015	5887	6900	7169	3804	6422	12125	9172	10751	9188	4695	12175	8243	10556	4962	6164	6300	4736	10018	7042	7427	8514	4834	7464	5895	6746	8063	5478	3580	6614	8269	6729
AMPL84984	*HRAS*	chr11:534256-534309	54	3975	7690	4844	0	10240	0	3746	4646	3947	2854	3841	3520	3526	4005	4164	2812	3773	6224	5518	5564	5082	2591	5590	3339	4221	3281	3021	3814	3276	5142	3898	3511	5026	2819	3860	2462	2793	3488	1988	1537	2689	3199	3846
AMPL82662	*ATM*	chr11:108117818-108117865	48	413	463	461	0	196	0	863	1077	866	784	916	870	948	1317	1217	607	1090	785	921	820	726	838	1141	1146	1692	592	1026	594	482	3409	3744	3098	3851	3314	3060	3642	3678	2904	2546	2514	1978	3150	1518
AMPL82666	*ATM*	chr11:108119778-108119831	54	1216	1573	1313	0	903	0	1594	2118	1894	1280	1685	1618	2142	2309	1602	708	1563	1029	1392	1017	1143	1301	1586	1590	1771	924	1581	1472	1125	1660	4723	3131	4429	2854	4418	3189	3318	3404	2023	2673	2280	3407	1928
AMPL82758	*ATM*	chr11:108123522-108123581	60	592	727	611	0	325	0	1022	1285	1064	895	1078	946	1200	1613	1353	836	1196	1081	1452	908	1019	1094	1303	1177	1545	583	1115	752	562	2001	2630	2047	3591	2546	3454	3213	4113	2352	1495	2240	1524	2675	1457
AMPL82011	*ATM*	chr11:108137942-108138015	74	1819	3515	2851	0	1473	0	3861	4531	3311	2622	3537	2956	3720	5163	2614	3463	3143	1756	2654	1959	1908	2088	2793	2389	3241	1513	2677	2226	1456	3601	3889	3581	5204	3507	4152	4674	4591	3504	3187	2644	2333	3843	2951
AMPL83554	*ATM*	chr11:108155126-108155179	54	4054	8267	8110	0	8121	0	5927	6771	5572	4459	5674	5216	5607	7324	4782	5168	5190	4847	6857	5348	4666	4940	6508	4850	6292	5268	5371	4142	3286	5036	7447	5517	6145	4424	5829	4835	5330	5109	4378	3820	3176	5698	5223
AMPL83653	*ATM*	chr11:108170456-108170513	58	1560	1834	2140	0	610	0	2857	3149	2409	2581	3014	2671	2939	3557	2485	2231	2753	2311	2524	2034	2297	2345	3076	2735	3144	1687	2227	2043	1312	3806	5249	4640	4787	3850	5028	3681	4052	3689	2609	3177	2605	4120	2758
AMPL83478	*ATM*	chr11:108172396-108172469	74	4469	8071	7835	0	6332	0	7213	7188	6331	5320	6309	5701	6289	9568	6606	7547	6339	8834	8545	8623	7024	5857	7832	8139	9873	7780	8903	7229	5995	7038	6411	6762	8397	5989	6537	5990	6643	6488	5877	4221	4153	7492	6613
AMPL83779	*ATM*	chr11:108173613-108173666	54	991	1657	1372	0	1070	0	1943	2243	2020	1270	1768	1621	1932	2143	1354	1204	1555	735	1296	885	872	963	1396	1269	1622	687	1120	1059	787	2624	3233	2358	3240	2565	2747	3075	2577	2784	2426	2017	1829	2797	1693
AMPL379514	*ATM*	chr11:108180928-108180995	68	452	1513	1611	0	833	0	741	711	556	388	539	479	605	1015	523	567	566	588	818	530	739	642	1509	894	1445	1967	1571	1198	1195	1368	1038	958	1407	816	1169	849	1023	728	751	701	585	958	870
AMPL84622	*ATM*	chr11:108200917-108200966	50	224	297	370	0	83	0	754	910	722	546	780	684	807	847	741	604	686	488	593	507	596	516	981	940	1243	538	809	703	375	1925	2823	1874	3051	1938	2712	2297	2741	1525	1362	1757	1204	1908	1059
AMPL143592	*ATM*	chr11:108204634-108204684	51	272	498	420	0	160	0	651	720	815	518	636	679	861	1032	639	629	702	394	616	393	497	523	640	684	883	363	615	496	297	1168	2395	1826	3013	1634	2701	2053	2734	1528	1172	1762	1482	1783	973
AMPL83501	*ATM*	chr11:108205763-108205828	66	2716	2510	3650	0	671	0	4986	5479	5506	4162	4512	4103	4560	5519	4765	283	4625	4827	5305	6123	5727	4307	8026	5315	5625	4890	5470	3742	3079	3298	5277	4802	4952	3948	4743	3762	4773	5477	3274	3126	3504	6416	4234
AMPL83530	*ATM*	chr11:108206582-108206636	55	1815	1669	2646	0	809	0	3465	4598	3565	2840	3215	2795	2821	3873	3341	3333	3367	3758	3358	4099	3862	3508	4601	3786	3740	2989	3315	2933	2819	3223	4503	4345	4462	3343	4967	2725	3440	3732	2451	2491	2685	4620	3188
AMPL83902	*ATM*	chr11:108218064-108218138	75	223	187	157	0	143	0	415	337	362	403	379	333	446	560	662	372	482	410	820	422	435	555	478	439	814	307	451	198	252	835	977	627	660	759	671	512	393	243	404	311	189	498	431
AMPL85006	*ATM*	chr11:108225560-108225634	75	657	859	637	0	134	0	1069	1185	1098	757	1156	1093	1584	1561	899	700	938	508	955	495	544	618	809	831	1172	359	835	564	315	2082	2549	1986	3044	2386	3077	4080	3723	2359	1949	2604	1629	2589	1343
AMPL84933	*ATM*	chr11:108236045-108236110	66	3105	7831	7608	0	3836	0	5856	5891	5471	3312	4995	4008	4537	6448	3231	3072	4099	2618	4612	2976	2823	3073	4172	3743	4399	2164	4672	3369	3039	6335	6108	4693	6626	4456	6020	7455	5796	4699	4746	3980	3393	5265	4394
AMPL85059	*ATM*	chr11:108236173-108236220	48	3201	4579	6611	0	5429	0	4060	5097	5351	3482	4065	3889	3500	4880	4662	3678	4046	6808	5777	6079	5703	5096	6142	4761	4480	3213	4239	3919	3934	3205	4868	3757	4229	2895	4868	2522	3289	4064	1916	2297	2805	4824	4100
AMPL88109	*KRAS*	chr12:25378521-25378590	70	253	171	305	0	101	0	1061	1502	1648	2345	1664	1448	1595	1352	1328	916	1856	729	448	987	934	930	1974	1692	1458	531	902	1222	872	1382	2337	2095	11103	1511	3067	2697	2674	1900	1166	1957	2440	2932	1607
AMPL87927	*KRAS*	chr12:25380258-25380338	81	2438	6744	6805	0	11814	0	5728	5737	6109	9539	5650	5105	5004	5594	5463	5888	5797	8618	4930	9119	7481	6316	10265	7513	8911	6654	6319	4153	3156	8786	6803	5692	21737	2946	5566	5322	5318	4171	5064	3732	5002	6352	6270
AMPL553293	*KRAS*	chr12:25398237-25398305	69	1426	6260	5986	0	32868	0	3289	4644	3717	6642	3370	3322	2934	3627	3327	3352	3426	5178	3710	4919	4597	3630	3167	2063	2948	3144	2166	1826	2355	2772	2088	1642	10228	1149	1631	1546	1859	1254	1371	1172	1601	1889	3764
AMPL309408	*PTPN11*	chr12:112888142-112888228	87	8006	22082	21728	0	30287	0	10773	11630	9474	7447	10596	9393	8131	9887	7834	10340	8526	10620	18772	11287	10043	8383	12384	10647	17713	13883	12050	8663	9874	19870	6851	8436	10624	7938	7419	9777	8884	6936	8407	5885	8325	9527	10698
AMPL86500	*PTPN11*	chr12:112926836-112926896	61	2444	7024	9383	0	3916	0	5717	6306	5232	4493	5693	5206	4794	5408	5005	5910	5840	7570	10364	7001	7085	4539	11229	7782	10947	11387	8949	6323	8659	16281	7007	7588	8854	6164	6614	6420	7007	5442	6212	4536	6162	7648	6670
AMPL87993	*HNF1A*	chr12:121431388-121431445	58	4508	5864	8516	0	7292	0	3722	6277	6226	4536	4648	4214	4358	4619	5218	3485	3638	5434	8610	7019	5601	3818	5197	4386	4707	3603	4891	3816	5114	3675	4519	4137	4000	3203	3522	2793	3591	3424	2364	2790	3367	4227	4403
AMPL507582	*HNF1A*	chr12:121432010-121432099	90	7925	18795	14325	0	16644	0	8318	9519	9829	6345	7638	7161	6487	8224	7571	8052	7330	10845	12554	10329	8923	6325	8343	9227	12720	5315	9886	6260	6588	13391	7247	7286	9925	7443	8144	7315	8370	5393	6443	5558	6746	7653	8390
AMPL87967	*FLT3*	chr13:28592607-28592663	57	5864	10220	10433	0	13424	0	5992	8234	6558	5879	5641	5993	5031	6677	5819	4754	6134	6839	8025	6489	5737	5321	6287	6355	7314	6770	6282	6006	6106	12896	7159	9149	10306	8579	8587	6068	7079	6682	5928	4610	6192	7134	6775
AMPL89417	*FLT3*	chr13:28602324-28602384	61	6968	12409	12880	0	16202	0	5810	8265	5632	7204	6096	7148	6096	7454	6763	6485	5826	7712	9898	9215	6954	6336	5972	6211	7323	7994	5723	5041	5265	12698	6088	8164	7626	7108	6691	4696	5494	5407	4466	4299	4895	6889	6891
AMPL89439	*FLT3*	chr13:28608212-28608289	78	2130	2228	1975	0	399	0	2279	3215	2879	2181	2540	2934	3317	3182	2374	2550	2714	1075	2058	1306	1349	1576	1599	1755	2272	1011	1502	1383	1031	5040	5583	5279	8024	5852	6847	6330	5976	4318	3521	4156	3956	4401	2955
AMPL90401	*FLT3*	chr13:28608332-28608386	55	1554	2285	2027	0	1803	0	1816	2652	2225	1839	2173	2343	2349	2468	2264	1395	2291	1510	1682	1499	1439	1660	1810	2393	1931	1028	1188	1520	1049	3856	4543	5261	6200	4867	5227	4304	4858	3813	2899	2591	3262	4067	2522
AMPL89861	*FLT3*	chr13:28610116-28610168	53	3429	7163	8830	0	7497	0	4942	5780	4308	5039	4554	4522	4153	5379	4239	3585	5081	5743	6822	5796	5882	4879	4947	4799	5706	5629	3934	3483	2892	13304	5446	7228	6768	4758	4956	3388	4012	3788	3482	2695	3761	4307	4926
AMPL91827	*RB1*	chr13:48919231-48919296	66	73	57	106	0	26	0	219	221	178	264	267	279	397	309	309	177	304	261	332	222	297	262	335	367	474	261	260	165	104	615	1460	1062	987	962	1028	936	1089	481	489	727	639	832	425
AMPL91829	*RB1*	chr13:48923136-48923198	63	147	185	157	0	120	0	293	270	230	463	389	333	566	524	596	325	514	473	692	343	478	554	675	592	998	352	452	205	283	1551	1313	1370	783	1139	785	600	487	271	571	345	301	518	506
AMPL255119	*RB1*	chr13:48942646-48942725	80	36	58	111	0	39	0	147	228	186	209	237	218	230	166	167	143	231	274	184	245	378	188	1216	1050	1003	965	591	712	452	1047	952	1197	1091	1112	817	1040	969	749	652	581	868	1017	518
AMPL91909	*RB1*	chr13:48955530-48955603	74	2289	4335	5376	0	2855	0	4763	5861	4617	4581	5013	4253	6162	5669	4038	2591	5638	3743	4414	3997	3812	3818	7927	5125	6734	6452	4651	4969	3674	7798	5685	6172	9710	6530	5544	6163	6503	5385	5352	4142	4397	6489	4934
AMPL416298	*RB1*	chr13:49027118-49027186	69	554	1014	1005	0	601	0	1437	1570	1325	1290	1669	1650	2534	1922	1100	1183	1736	686	987	736	963	760	2123	1825	2214	970	1180	1541	809	4615	4215	4983	7856	4576	3857	5966	4696	3170	3794	3233	3041	4271	2230
AMPL555556	*RB1*	chr13:49033827-49033903	77	4271	13952	11214	0	18305	0	6335	7241	5230	5627	5956	5289	6984	6582	4559	4370	6159	6340	9015	6788	5914	5335	7558	5310	8144	8484	5824	4356	3709	15272	4913	6166	8805	5312	4118	5275	5140	4319	5328	3094	3773	5141	6322
AMPL147815	*RB1*	chr13:49037846-49037932	87	134	165	168	0	48	0	275	263	226	370	344	387	586	399	466	279	372	249	406	259	305	310	464	401	604	304	346	181	176	1969	1822	1740	3610	1734	2062	2382	2452	965	898	1766	1024	1355	768
AMPL91174	*RB1*	chr13:49039149-49039216	68	671	673	647	0	360	0	746	992	715	809	848	1013	1741	1102	945	644	984	564	874	608	649	748	835	761	1042	469	562	445	340	2530	4768	4218	7792	4535	5156	4108	4969	2248	2015	3900	2095	2799	1712
AMPL329410	*AKT1*	chr14:105246406-105246502	97	10116	14258	21006	0	2516	0	9704	11275	9429	7789	9381	8867	8759	8430	8789	6510	11824	10992	10562	13418	11755	12339	12525	9044	11987	8636	6348	7824	6231	12600	7466	8298	9665	7204	7855	8641	7823	5730	6660	5672	5585	9100	8872
AMPL90912	*CDH1*	chr16:68835616-68835684	69	11593	23580	21621	0	77545	0	10467	11798	9286	7936	8769	8405	9360	11893	10200	6747	8546	11097	13141	12871	10163	9552	11350	9418	12043	16995	8609	7910	7061	26977	9106	9295	10293	9755	8281	7322	9805	5939	6056	4371	7981	9024	11718
AMPL91050	*CDH1*	chr16:68846080-68846148	69	4853	5621	8914	0	6464	0	5513	6870	5936	4550	5630	4691	4589	6105	5826	4851	5111	8261	7303	8435	7606	7077	7358	6767	7023	7466	5202	5311	4734	8130	4990	6345	5848	5252	5422	4273	5381	4547	3391	2578	5634	6142	5619
AMPL91406	*CDH1*	chr16:68847263-68847322	60	4611	9094	11524	0	22124	0	5657	6762	5900	4038	5139	4527	4433	6099	4978	4341	4273	7104	8617	7378	5781	5662	7311	5145	7100	8995	4471	3700	3356	12299	4629	4773	5413	5223	4136	4388	4423	3146	3676	2071	4596	4616	5750
AMPL110679	*TP53*	chr17:7573997-7574069	73	2124	5024	4958	0	2846	0	4669	5612	4923	3743	5178	4291	2409	4187	4305	4068	4343	6327	4521	7146	5658	7058	7321	7752	8998	5107	4297	5428	4872	9632	5331	6131	5996	4079	5503	5272	5913	4699	4827	5410	4938	6763	5040
AMPL117792	*TP53*	chr17:7577021-7577131	111	3420	6227	5274	0	2577	0	6336	6493	6550	6292	7474	6187	3849	6293	6737	5622	6917	10258	6463	5302	9214	11125	8600	8912	12051	5236	5712	6230	4009	11235	5981	6356	6125	5820	7704	8362	6535	6733	5386	6708	6443	8414	6456
AMPL94170	*TP53*	chr17:7577522-7577591	70	2668	4464	6294	0	1328	0	4982	7127	4853	4447	5122	4971	2721	5226	5059	4714	4904	6801	6284	7365	6426	8131	6577	4586	6872	5249	3663	5278	5135	7903	6075	5283	5840	3390	4824	4468	5041	4463	2968	3609	3199	6103	4867
AMPL226789	*TP53*	chr17:7578180-7578288	109	4717	12399	8976	0	10307	0	7120	7806	6179	5078	6464	6052	3088	6252	5591	5403	6121	9282	5859	9027	7978	9753	6780	6368	9044	4428	4606	5662	4929	7040	4718	5781	5905	3899	6507	5312	5959	5006	3174	5040	4479	5689	6042
AMPL226369	*TP53*	chr17:7578387-7578483	97	1360	1974	2397	0	523	0	2122	3048	2475	2142	2326	2337	1208	2071	2629	1788	2606	4460	2035	4289	3741	4209	4058	3471	4026	2254	2227	2997	3102	1787	3087	3551	3078	1624	3221	1834	2564	2952	1534	2512	2592	3872	2526
AMPL658417	*TP53*	chr17:7578525-7578579	55	4066	10777	8119	0	17885	0	5434	6899	5779	3994	5222	5232	2746	5185	5998	4578	5184	8586	6362	8241	6463	8647	6278	5269	6399	3435	3089	3419	3213	3957	4294	4886	4747	2956	4211	3628	4541	3550	3465	3155	3277	5266	5201
AMPL484618	*TP53*	chr17:7579345-7579445	101	4316	9449	5701	0	13697	0	4223	5109	5367	4430	5110	4238	2832	4904	5606	3696	5064	8295	5260	8540	7032	9405	5373	5069	6075	2601	2936	3435	2123	4529	4834	4387	3428	2225	4449	3727	3737	3885	2394	3591	3218	5479	4756
AMPL310032	*TP53*	chr17:7579859-7579941	83	2657	6147	4614	0	4384	0	3963	5242	5023	3470	4829	4635	2849	3425	4974	2786	3654	8732	4601	8787	6471	8797	6817	6314	6627	2888	3920	3963	3625	7984	5302	4655	4345	2698	4650	3813	4710	4587	3075	4498	4113	4986	4610
AMPL484410	*ERBB2*	chr17:37880213-37880284	72	234697	13516	8475	0	19067	0	5475	7102	6710	5544	5169	5702	7068	5831	5825	3315	5464	7392	9092	6990	6421	7839	8315	4705	5475	4628	9172	2824	1912	8945	4095	3268	5216	76311	4047	3171	3528	7437	2681	2481	6286	4138	12984
AMPL93379	*ERBB2*	chr17:37880987-37881061	75	157157	5733	7012	0	7376	0	3703	5901	5072	4846	4220	4922	5278	4428	5307	3481	4546	8917	5800	8664	7474	7443	9709	5867	6015	4962	10577	4717	4374	5217	5692	5131	6635	73551	5258	3198	4100	10337	2937	3962	8978	6969	10844
AMPL92464	*ERBB2*	chr17:37881297-37881364	68	183054	7212	7348	0	9333	0	4289	5084	4897	5380	4559	5726	6647	4340	5435	2891	5141	4900	6248	7265	5607	6509	8788	5174	3997	5252	9569	4486	3819	4948	5996	4610	5829	63830	4548	2873	3520	6846	2431	3796	8422	5419	10858
AMPL504796	*ERBB2*	chr17:37881433-37881497	65	140868	7808	7023	0	11117	0	4390	5686	5645	4831	4222	5006	5779	4159	4950	3114	5469	7911	7250	9190	5905	7593	7298	3917	4669	4729	7869	2972	2870	4462	4377	4630	4922	69593	3760	3006	3066	7614	2305	2883	5750	5087	9850
AMPL98573	*SMAD4*	chr18:48575133-48575188	56	233	192	173	0	84	0	439	612	391	568	420	360	326	697	872	490	568	626	991	587	631	373	427	813	1019	290	567	332	251	1033	2123	1519	2111	228	1748	1209	2093	564	861	1680	743	1588	734
AMPL98681	*SMAD4*	chr18:48581197-48581246	50	1956	2752	3201	0	1291	0	2358	3589	3161	2853	2579	2248	1571	3205	3194	2725	2949	6244	5903	5134	4541	2519	2993	4241	4825	2495	4267	3246	2651	3000	4223	3911	3697	439	3701	2479	3219	2326	2562	2527	2461	4368	3038
AMPL99638	*SMAD4*	chr18:48584511-48584567	57	4755	8722	8821	0	6490	0	5396	7563	6584	4964	4742	4232	3053	6459	5743	4555	5630	8701	11408	7799	7047	3725	4033	5759	7330	4673	6463	5556	3825	5539	6325	5007	5447	547	5482	4132	4496	3413	3969	3949	3374	6225	5284
AMPL552959	*SMAD4*	chr18:48586238-48586305	68	2018	5542	4017	0	4256	0	2971	4435	3952	3350	3174	2771	1944	3882	3297	2356	3761	3708	6061	3927	4138	2071	1701	2214	2517	1552	2205	2266	1377	1531	3085	2325	2541	195	2324	1583	2074	1487	1527	1555	1352	2639	2612
AMPL121852	*SMAD4*	chr18:48591825-48591924	100	2503	3329	3446	0	1089	0	4347	5008	4210	4846	5016	3801	2515	5067	4422	4309	4995	6958	8620	8467	7315	3028	5452	8933	9282	4357	7243	5334	2071	8421	4611	5271	5833	663	5914	6567	6357	4546	4762	5562	4688	7267	4915
AMPL516350	*SMAD4*	chr18:48593402-48593459	58	290	287	497	0	144	0	658	939	982	936	709	772	417	737	1330	493	934	2786	1384	2179	1774	901	1726	2648	1857	1103	1401	1245	690	1107	2185	1859	1438	174	1280	986	1406	1022	1133	885	954	2173	1105
AMPL101246	*SMAD4*	chr18:48603031-48603113	83	3374	5300	4813	0	4295	0	3317	4610	4020	3897	3504	3035	2094	3857	4514	3109	4052	6910	6475	6737	5217	2786	3638	5221	5057	2711	3879	3531	2492	3414	5078	4912	3775	339	4229	2491	3467	2974	2496	2982	2696	5432	3732
AMPL116963	*SMAD4*	chr18:48604667-48604755	89	5643	12235	10672	0	6303	0	9164	9938	8843	7061	7040	5509	3968	7844	7765	7176	7231	10745	15953	8452	10806	5276	7985	13065	18437	7702	13760	9144	4990	21939	9666	9889	12240	1219	10519	11146	10934	6903	10943	8229	6906	12353	8943
AMPL482833	*STK11*	chr19:1207000-1207098	99	9510	31814	17090	0	56941	0	11095	10469	9798	8634	10135	9836	5905	10271	9801	9662	8723	11827	14072	13037	10742	6969	10410	9897	13003	7769	7229	6578	4893	12937	9080	8135	8770	6557	8318	8567	9434	6203	7118	4675	6792	10501	10552
AMPL503320	*STK11*	chr19:1220427-1220502	76	4111	8193	7258	0	8925	0	5601	7296	6550	2869	5521	4946	3329	5666	6800	5629	6272	8116	7456	8296	6697	4695	9165	6652	6837	5049	3987	5714	4773	5330	6514	6716	5836	3986	5850	3984	5200	5185	3537	2605	4881	7712	5565
AMPL103879	*STK11*	chr19:1221236-1221320	85	1248	863	2216	0	234	0	6422	6879	6063	5178	6989	6222	2784	5939	6415	1198	6784	6242	6921	7962	5156	4732	12383	10304	7671	7444	6651	7704	6182	11557	5311	7458	8358	7008	5617	8951	6512	6767	6738	4512	8013	10235	5996
AMPL490549	*STK11*	chr19:1223036-1223141	106	6792	24119	12326	0	30864	0	8225	8831	7431	6469	7865	7317	3926	8086	7384	6941	6946	9658	10870	10895	8178	5607	7113	6014	6941	3873	3765	5050	3880	6064	4580	4631	5701	3989	4736	5004	5201	4247	3658	2517	4544	4899	7027
AMPL103227	*JAK3*	chr19:17945675-17945734	60	1140	1169	1928	0	270	0	2332	3056	2576	2321	2471	2229	2141	2300	2861	601	3088	4734	3443	4424	3666	2658	6125	4866	4520	2382	2842	2913	2241	3474	4339	4253	4483	2758	3639	2857	3432	3149	2259	1723	3366	3915	2832
AMPL103630	*JAK3*	chr19:17947992-17948062	71	5625	8452	9418	0	8282	0	6046	7061	5589	5056	6222	4979	3004	6622	6481	5099	7206	10884	8825	8938	8707	5936	9121	7801	10534	3925	5810	5170	4413	9066	6235	6869	7395	4661	5828	4652	6138	4662	4939	2967	5176	7933	6232
AMPL649396	*SRC*	chr20:36031712-36031771	60	9145	24064	19279	0	60780	0	10000	9343	10571	6510	7503	7935	10469	10397	7229	5552	7701	9914	11652	11141	8293	14760	10791	6009	8476	8296	11715	4908	4223	16847	5922	5783	7486	4736	6014	4598	5790	6408	4690	3939	7187	5951	9572
AMPL104671	*GNAS*	chr20:57484403-57484471	69	2339	2702	4344	0	1429	0	3727	3994	4227	3082	3716	4061	4245	4498	3069	3010	3893	6653	5764	5610	5763	6965	1218	906	1002	1120	1022	668	513	1017	823	760	927	652	625	686	696	582	503	706	944	794	2363
AMPL104720	*SMARCB1*	chr22:24133965-24134014	50	4872	10167	13621	0	10276	0	6971	8509	6832	5992	7074	6110	6099	9477	6035	3800	6858	7830	8543	9246	7739	5518	12291	7976	9707	9920	7829	6901	5245	10609	8772	9299	7445	5442	7652	5398	6269	5899	5594	3674	6108	7724	7174
AMPL107255	*SMARCB1*	chr22:24143229-24143298	70	6729	12485	11860	0	10352	0	7411	8946	8510	6669	8082	7441	7815	9689	8821	6467	7308	8636	11355	10317	8185	7548	14562	9119	10168	8387	7811	7862	6307	10003	7835	7054	7227	5800	6587	5602	7974	5808	4766	3619	6966	8437	7774
AMPL106398	*SMARCB1*	chr22:24145525-24145589	65	5591	10090	13700	0	7029	0	6289	8151	5940	5551	6921	6227	5745	7692	6557	5126	6911	8662	8628	8700	7346	5487	9815	7076	7814	8945	6828	6698	7011	6771	6959	7008	6259	4659	6628	4143	5837	4941	3877	3067	5559	6710	6499
AMPL484088	*SMARCB1*	chr22:24176335-24176402	68	5190	6400	9701	0	9815	0	4603	6934	5851	4253	5045	4402	4409	6490	5308	2181	4106	7653	6253	8386	6042	4576	7771	4350	4092	4777	4372	4314	3440	3323	4028	4529	3948	2731	3915	2745	3006	2863	2667	2123	3699	4763	4644

[a] Samples with 10% or more amplicons of low coverage

Supplementary Table S8. The Coverage of an individual region in 32 out of 74 gastric cancers analyzed in this study

Amplicon	Gene	Region	Length	B-GC58T	B-GC60T	B-GC61T	B-GC62T	B-GC63T	B-GC64T	B-GC66T	B-GC68T	B-GC70T	C71T(1/1(	B-GC73T	B-GC74T	B-GC75T	B-GC77T	B-GC78T	B-GC80T	B-GC81T	B-GC82T	B-GC83T	B-GC85T	B-GC86T	B-GC87T	B-GC88T	B-GC90T	B-GC91T	B-GC92T	B-GC95T	B-GC96T	B-GC97T	B-GC98T	B-GC99T	B-GC72T	Average
AMPL495041	*MPL*	chr1:43814962-43815050	89	1448	1115	1067	991	1165	1186	824	880	749	1017	853	865	790	593	758	927	1000	800	1024	758	1007	741	943	1150	976	1846	1328	1506	1436	1390	1205	1516	1058
AMPL30014	*NRAS*	chr1:115256504-115256554	51	1014	890	630	801	779	814	610	588	617	779	601	645	724	970	563	675	837	533	749	457	780	545	586	532	597	926	789	764	616	762	779	1086	720
AMPL30373	*NRAS*	chr1:115258717-115258765	49	1332	1125	1000	1011	1103	1128	697	882	889	1053	1038	928	955	1295	689	857	995	678	830	620	1180	669	968	836	978	1499	1156	1215	988	1190	1207	1696	1021
AMPL34014	*ALK*	chr2:29432657-29432711	55	1726	1681	1143	1227	1433	1424	1082	1280	1181	2276	1013	1810	1292	1994	1441	1141	1191	1032	1235	1090	1019	985	1092	1236	1350	1674	1459	1333	1045	1638	1259	2499	1384
AMPL33013	*ALK*	chr2:29443688-29443741	54	994	818	609	308	426	392	516	642	559	1325	576	34	711	901	625	311	702	607	790	248	775	588	705	396	233	534	988	964	394	853	437	1387	636
AMPL31381	*IDH1*	chr2:209113105-209113153	49	978	730	728	706	801	818	564	629	587	958	623	920	750	969	757	709	724	550	648	838	1025	717	711	879	676	1201	1106	1074	874	975	1100	1672	844
AMPL31533	*ERBB4*	chr2:212288919-212288978	60	691	745	510	510	669	641	471	567	499	666	361	586	430	617	548	488	583	442	516	351	608	466	508	623	570	917	750	668	496	630	618	733	577
AMPL226460	*ERBB4*	chr2:212530063-212530155	93	1576	1707	1183	1449	1690	1503	1268	1498	1325	1733	885	1530	1381	1424	1348	1104	1314	1086	1551	749	1517	1036	1081	1489	1112	1662	1614	1723	1277	1562	1284	1814	1390
AMPL33659	*ERBB4*	chr2:212576812-212576872	61	664	670	469	524	548	678	466	594	535	751	417	543	563	685	601	567	600	522	613	333	709	488	506	617	497	807	723	734	592	712	641	1130	609
AMPL645344	*ERBB4*	chr2:212578292-212578350	59	917	921	719	900	830	918	670	650	739	936	629	769	647	965	816	686	845	606	595	595	675	565	642	632	577	922	693	743	698	618	709	1303	754
AMPL32827	*ERBB4*	chr2:212587136-212587185	50	952	977	713	869	1035	1043	635	701	685	1222	700	754	881	1137	904	850	1041	722	737	606	915	755	647	843	823	1010	869	931	860	967	971	1947	897
AMPL33781	*ERBB4*	chr2:212589768-212589840	73	1524	1247	1041	1128	1415	1344	1025	1164	1042	1625	923	1498	1115	1201	1131	1092	1285	998	1198	671	1563	1169	1098	1335	1253	2041	1657	1648	1498	1587	1369	1944	1307
AMPL32781	*ERBB4*	chr2:212652717-212652775	59	313	267	129	192	156	256	296	367	551	457	262	453	452	1139	806	716	738	391	290	380	194	251	281	396	249	557	637	602	541	646	608	471	439
AMPL33555	*ERBB4*	chr2:212812107-212812169	63	413	289	168	141	194	238	325	443	577	449	280	360	351	784	706	444	482	415	441	454	298	322	352	417	340	492	395	439	455	412	463	442	399
AMPL613072	*VHL*	chr3:10183795-10183859	65	1154	1015	793	729	1109	876	756	754	792	977	810	876	634	608	791	868	978	621	791	834	802	594	861	910	591	1253	913	1091	816	975	909	1481	874
AMPL232121	*VHL*	chr3:10188216-10188302	87	1540	1660	1096	1088	1767	1381	1244	1103	1197	1405	1027	1276	989	1037	1043	1227	1347	810	969	1136	1055	764	969	1152	798	1850	1327	1414	1133	1690	1177	2067	1242
AMPL34860	*VHL*	chr3:10191478-10191532	55	1574	1335	1113	1131	1722	1344	1211	1077	1137	1431	1034	1223	806	1042	1125	1311	1317	861	1040	1240	1071	814	1039	1159	899	1878	1513	1467	1216	1393	1397	1970	1247
AMPL38772	*MLH1*	chr3:37067208-37067266	59	1868	1967	1353	1503	1162	1664	1475	1248	1491	1506	1188	1215	830	1257	1101	1455	1723	979	1298	1074	1264	827	1064	1233	861	1567	1378	1381	1142	1430	1264	2127	1340
AMPL423052	*CTNNB1*	chr3:41266039-41266137	99	2034	1791	1300	1271	1353	1717	1536	1404	1228	1616	1045	1529	1164	919	923	1540	1566	1169	1248	911	1903	1006	1241	1622	1038	2161	1968	2142	1644	2234	1565	2040	1495
AMPL36101	*PIK3CA*	chr3:178916844-178916907	64	870	724	511	813	771	697	534	458	469	509	624	390	414	589	593	477	487	432	532	614	335	285	455	478	359	632	373	584	460	406	452	787	535
AMPL554194	*PIK3CA*	chr3:178921502-178921570	69	711	431	407	402	536	495	492	480	565	428	433	417	362	697	522	448	448	389	381	815	457	340	559	459	425	607	536	575	636	559	502	1001	516
AMPL36569	*PIK3CA*	chr3:178927931-178927986	56	239	139	90	180	147	158	208	195	281	219	187	194	223	560	393	354	371	174	172	208	99	88	173	156	93	431	397	364	342	456	414	277	249
AMPL35831	*PIK3CA*	chr3:178936057-178936111	55	1331	1123	857	1244	1244	1134	700	750	874	828	931	720	809	1472	1057	995	1064	730	699	1513	725	638	1050	728	660	1086	747	846	887	1020	785	1779	970
AMPL339432	*PIK3CA*	chr3:178938822-178938906	85	491	388	292	319	349	334	523	559	484	531	211	397	446	484	362	368	393	226	196	346	217	217	258	251	193	633	440	490	388	586	407	527	385
AMPL118631	*PIK3CA*	chr3:178952002-178952091	90	575	500	408	543	620	463	474	515	515	546	419	434	350	504	431	485	464	258	260	734	358	322	569	465	458	514	459	564	676	885	651	982	512
AMPL37385	*PIK3CA*	chr3:178952139-178952190	52	1359	841	750	1000	1050	1117	823	833	708	803	842	729	850	1162	866	884	719	949	1024	942	774	659	984	900	860	1286	999	1141	913	1054	1027	2004	964
AMPL508777	*FGFR3*	chr4:1803549-1803626	78	917	614	691	507	637	534	469	594	503	558	723	536	397	348	694	530	534	339	479	561	567	429	705	609	472	869	713	754	759	863	676	981	611
AMPL142574	*FGFR3*	chr4:1806111-1806208	98	2069	1399	1631	1207	1538	1469	1167	1326	1142	1456	1197	1406	1053	726	1499	1294	1348	1004	1577	1091	1702	1095	1855	1911	1503	2589	2433	2686	1999	2509	1783	1848	1579
AMPL411633	*FGFR3*	chr4:1807841-1807931	91	1332	919	1067	792	894	890	702	810	774	852	955	776	735	451	1068	842	974	646	713	694	843	582	1046	1045	750	1301	1147	1374	901	1327	938	1387	923
AMPL493041	*FGFR3*	chr4:1808283-1808378	96	2121	1680	1921	1372	1767	1502	1391	1413	1443	1640	1281	1480	1171	935	1942	1721	1663	1151	1548	1414	1573	1061	1972	1781	1436	2383	2312	2223	2007	2580	1810	2062	1680
AMPL505293	*FGFR3*	chr4:1808938-1809007	70	1392	1007	1059	827	1030	995	771	960	781	1037	856	874	866	588	1086	1048	856	682	1024	720	992	785	1251	1214	940	1708	1462	1470	1106	1562	1275	1596	1057
AMPL43181	*PDGFRA*	chr4:55140988-55141063	76	1203	1302	934	1304	1242	1173	926	971	984	1180	791	956	1146	853	874	922	1159	780	1016	781	1018	811	697	1014	865	1412	1284	1179	1202	1237	1105	1985	1072
AMPL43383	*PDGFRA*	chr4:55144112-55144180	69	927	908	725	868	837	891	706	801	827	956	507	860	848	662	672	766	889	615	755	734	991	813	618	913	889	1253	1200	1250	1117	1060	1044	1650	892
AMPL43200	*PDGFRA*	chr4:55144521-55144588	68	1558	1506	1124	1577	1524	1479	1190	1312	1458	1372	835	1088	1251	1438	1280	1305	1549	1008	1173	1301	1120	1019	849	1276	1077	1604	1426	1425	1471	1396	1410	2396	1337
AMPL234145	*PDGFRA*	chr4:55152023-55152109	87	1876	1677	1537	2181	2146	1901	1518	1779	1789	1756	1354	1628	1869	1480	1611	1788	2164	1284	1706	1699	1677	1625	1316	1823	1575	2418	2381	2368	2176	2130	1950	3781	1874
AMPL44733	*KIT*	chr4:55561749-55561816	68	1336	1306	1057	1308	1253	1281	917	1044	1156	1145	965	1057	1020	902	954	867	1205	829	882	767	809	737	685	1140	849	1370	1180	1187	1047	1027	1074	2025	1075
AMPL46436	*KIT*	chr4:55592147-55592230	84	155	167	98	149	110	149	144	153	216	179	112	156	169	263	221	226	256	151	137	134	96	97	96	122	73	395	407	376	332	354	409	209	197
AMPL46166	*KIT*	chr4:55593449-55593509	61	710	495	343	423	423	559	570	678	780	699	377	451	619	668	592	611	572	661	833	372	541	441	438	562	476	851	749	740	656	557	800	964	600
AMPL178642	*KIT*	chr4:55593582-55593689	108	207	192	165	234	235	163	165	228	225	241	106	174	158	159	203	219	255	139	173	194	141	135	100	181	189	280	219	258	258	345	372	368	209
AMPL47704	*KIT*	chr4:55594220-55594294	75	1524	1463	1266	1756	1627	1660	1230	1360	1436	1567	910	1080	1295	1219	1188	1370	1529	1094	1410	968	1176	1004	841	1285	1056	1533	1437	1373	1292	1411	1377	2079	1338
AMPL48478	*KIT*	chr4:55595496-55595562	67	1237	971	790	1148	1059	1050	837	945	1207	949	641	681	884	986	1091	875	995	892	906	938	607	693	690	908	710	1207	944	1005	950	694	1087	1843	951
AMPL47725	*KIT*	chr4:55597468-55597535	68	1208	1147	922	1083	1170	1061	866	1015	903	1035	782	1003	1021	729	763	936	952	886	1016	554	1080	822	794	1206	1098	1637	1511	1477	1219	1159	1372	1893	1072
AMPL49160	*KIT*	chr4:55599295-55599365	71	799	980	654	883	853	805	595	650	633	782	513	696	769	678	605	667	742	708	853	382	691	579	558	805	631	1042	983	971	673	684	827	1258	748
AMPL49191	*KIT*	chr4:55602686-55602740	55	794	706	664	833	858	757	553	726	677	810	547	698	815	554	572	653	693	577	777	391	676	625	542	771	696	1072	1078	1046	749	761	986	1342	750
AMPL49305	*KDR*	chr4:55946111-55946171	61	1037	855	791	892	910	883	662	863	678	854	561	819	772	467	575	717	725	671	929	377	925	751	744	1205	953	1390	1322	1486	842	877	1206	1180	872
AMPL49402	*KDR*	chr4:55946218-55946277	60	1620	1713	1410	1760	1692	1796	1193	1378	1231	1764	1115	1376	1585	1269	1300	1596	1854	1167	1227	1248	1342	984	1040	1483	1267	1982	1864	1616	1563	1694	1767	2794	1521
AMPL50673	*KDR*	chr4:55953790-55953860	71	1536	1476	1176	1559	1469	1577	1123	1328	1263	1300	771	1171	1229	961	1161	1201	1494	1003	1255	865	1083	944	827	1282	1003	1564	1595	1353	1244	1382	1417	1710	1260
AMPL50695	*KDR*	chr4:55955062-55955132	71	1458	1177	1083	1354	1308	1309	984	1162	967	1298	795	1113	996	869	1009	1154	1367	966	1161	932	1102	985	880	1250	1061	1664	1385	1365	1446	1246	1397	1935	1193
AMPL52170	*KDR*	chr4:55960987-55961064	78	1961	2113	1617	2058	1897	1992	1464	1611	1688	1789	1097	1418	1646	1428	1638	1608	2258	1271	1447	1578	1418	1157	1067	1589	1222	2091	2115	1834	1752	1786	1631	2698	1686
AMPL52189	*KDR*	chr4:55962450-55962522	73	1439	1709	1026	1522	1440	1549	1144	1171	1159	1290	738	1088	1194	980	1151	1104	1526	874	1103	1003	1011	796	707	1081	958	1413	1223	1227	1028	1225	1075	1771	1179
AMPL52386	*KDR*	chr4:55972921-55972974	54	1009	1144	860	1114	1010	904	666	828	821	845	606	676	744	654	858	768	947	707	799	602	688	572	453	748	667	921	817	785	696	655	813	1102	796
AMPL52394	*KDR*	chr4:55979608-55979656	49	1225	935	860	1062	1015	1061	827	777	943	939	722	818	947	1101	1062	878	1022	822	829	854	758	691	674	904	808	1136	1020	1014	954	852	1163	1941	957
AMPL52210	*KDR*	chr4:55980318-55980372	55	697	612	441	543	534	591	520	492	583	582	345	438	475	502	523	498	500	492	606	415	394	371	421	607	445	744	572	581	492	436	635	756	526
AMPL40184	*FBXW7*	chr4:153245442-153245493	52	992	924	810	731	860	893	655	752	699	819	948	767	766	655	485	772	756	708	627	371	732	559	566	549	726	1123	906	992	829	803	945	1588	791
AMPL40234	*FBXW7*	chr4:153247285-153247369	85	1487	1531	1140	1120	1461	1409	1088	1232	974	1452	935	1164	1231	702	686	1356	1337	994	1320	464	1378	940	961	1106	1240	1917	1617	1910	1497	1718	1467	2040	1277
AMPL41691	*FBXW7*	chr4:153249363-153249429	67	1225	995	897	969	1102	1080	851	940	995	1061	890	845	944	796	646	1109	883	683	948	398	787	585	699	628	786	1028	836	1115	738	759	891	1575	896
AMPL41812	*FBXW7*	chr4:153250860-153250918	59	786	752	554	583	738	758	573	676	695	749	471	431	643	686	519	730	787	476	555	356	587	462	448	484	531	809	735	728	718	822	721	1480	658
AMPL42297	*FBXW7*	chr4:153258979-153259025	47	508	393	309	262	351	365	224	312	276	302	385	207	328	266	255	317	288	339	329	113	326	246	223	269	271	423	268	425	256	214	393	521	311
AMPL53695	*APC*	chr5:112173884-112173947	64	1322	1384	1079	1110	1460	1382	1010	1099	1179	937	1005	948	898	986	871	1084	1289	920	978	893	1337	779	773	1120	888	1495	1315	1199	1239	1327	1272	2028	1144
AMPL54189	*APC*	chr5:112174605-112174655	51	689	699	541	587	696	711	517	539	535	558	491	470	500	568	442	614	652	599	659	415	756	461	492	663	491	825	816	764	686	733	807	1168	630
AMPL320120	*APC*	chr5:112175158-112175255	98	1119	1024	864	838	1209	1051	818	983	909	743	675	906	724	625	557	813	773	783	1025	583	1496	701	764	1219	1025	1422	1511	1607	1208	1160	1232	1304	990
AMPL227540	*APC*	chr5:112175337-112175435	99	1123	873	719	742	1043	1012	681	853	609	735	720	837	732	426	431	735	777	778	1021	517	1565	794	779	1238	1063	1482	1606	1789	1332	1293	1275	1449	970
AMPL145156	*APC*	chr5:112175581-112175690	110	810	983	716	719	964	858	720	833	697	596	467	681	546	618	566	735	892	586	834	561	1045	572	566	891	648	919	1051	950	976	1128	903	980	782
AMPL59934	*APC*	chr5:112175752-112175816	65	1421	1355	1104	1117	1447	1457	1001	1055	1187	1043	1064	983	867	1181	892	1113	1364	975	965	1028	1296	814	808	1067	874	1308	1287	1114	1260	1254	1308	2327	1167
AMPL60049	*APC*	chr5:112175938-112175982	45	105	50	46	36	42	70	73	88	112	67	98	66	71	126	107	99	117	111	93	77	65	51	77	135	61	144	109	128	88	111	128	137	90
AMPL60239	*CSF1R*	chr5:149433629-149433695	67	2103	1633	1264	1324	1499	1625	1150	1368	1268	1062	1030	1229	1022	978	942	1222	1592	1051	1342	1200	1346	1010	1021	1413	1196	2058	1713	1539	1598	1579	1606	2527	1391
AMPL59674	*CSF1R*	chr5:149452992-149453051	60	1392	929	834	790	913	967	575	716	712	719	561	775	688	569	496	756	868	719	798	626	1071	698	684	1076	968	1531	1437	1510	1104	1176	1162	1521	917
AMPL236158	*NPM1*	chr5:170837502-170837599	98	576	404	411	358	570	517	368	378	326	267	342	307	355	240	258	315	291	492	467	204	554	256	306	514	422	736	549	665	312	332	372	558	407
AMPL307725	*EGFR*	chr7:55211024-55211132	109	2396	1978	1995	1721	2577	2347	1585	1638	1739	2105	1446	2470	1725	2097	1611	1738	2038	1433	1729	1473	1535	1404	2035	1569	1120	2499	1836	2448	2142	2794	2122	2478	1932
AMPL68178	*EGFR*	chr7:55221796-55221866	71	1067	881	808	724	1240	876	739	877	657	1015	813	1218	941	716	584	669	753	670	867	441	516	884	1391	1119	818	1723	1222	1892	977	657	1454	1193	950
AMPL1594224	*EGFR*	chr7:55232962-55233053	92	2786	2546	2779	2196	3295	2885	2059	2089	2184	3048	1754	3107	2221	2828	1759	2078	2732	1654	1936	2072	1893	1797	2424	2017	1311	3455	2408	2842	2774	3371	2644	3375	2447
AMPL67641	*EGFR*	chr7:55241644-55241717	74	1274	1095	964	943	1131	1245	722	981	762	1148	906	1216	1060	790	645	681	779	795	966	462	924	776	1313	993	611	1633	1152	1584	955	948	1142	1226	994
AMPL1598122	*EGFR*	chr7:55242416-55242540	125	1757	1536	1535	1243	2080	1644	1031	1267	1012	1300	851	1710	1027	917	1108	1077	1324	1118	1409	740	1212	1023	1630	1325	857	2186	1524	2089	1491	1782	1539	898	1351
AMPL493236	*EGFR*	chr7:55248999-55249072	74	527	470	504	390	542	522	349	437	330	614	474	690	432	367	286	350	366	360	498	223	705	546	837	681	496	1028	881	1386	615	693	875	729	569
AMPL174184	*EGFR*	chr7:55249109-55249187	79	2158	2128	2070	1629	2474	2188	1561	1590	1586	2282	1453	2493	1187	1971	1452	1616	1978	1245	1528	1370	1491	1410	2162	1700	1083	2685	1892	2236	2162	2599	2092	2700	1880
AMPL1599089	*EGFR*	chr7:55259432-55259539	108	2660	1450	2223	2012	2937	2503	2116	1689	1864	2467	1545	2753	2244	2275	1967	1922	2430	1584	2077	1662	1743	1682	2469	1992	1273	2877	2374	2604	2530	3381	2259	2501	2190
AMPL61096	*MET*	chr7:116339618-116339697	80	1538	1488	1426	1288	1665	1492	966	1141	1010	1723	906	832	1344	1289	898	1126	1331	965	1345	695	1106	1253	772	1173	6646	1809	1410	1732	1237	1193	1477	1726	1438
AMPL61118	*MET*	chr7:116340222-116340275	54	1306	1171	1178	921	1391	1225	859	1057	879	1499	876	791	1323	1430	768	911	992	845	993	639	1000	1172	793	1101	6872	1606	1436	1592	1247	1162	1702	2274	1344
AMPL62593	*MET*	chr7:116411935-116412010	76	1592	1302	1509	1174	1762	1473	1002	1170	999	1530	978	852	1290	1315	954	1077	1216	915	1102	753	955	1068	727	1035	6467	1358	1258	1672	1176	1086	1531	1758	1377
AMPL62615	*MET*	chr7:116417428-116417506	79	2033	1960	1950	1610	2477	1906	1339	1619	1504	1953	1178	1147	1568	2165	1384	1387	1782	1079	1253	1269	1191	1330	969	1336	8127	1641	1567	1774	1641	1544	1628	2467	1806
AMPL64212	*MET*	chr7:116423412-116423475	64	1831	1606	1685	1506	2062	1821	1308	1329	1395	1827	1099	962	1379	2100	1209	1364	1570	981	1232	1193	1105	1265	865	1230	7173	1530	1470	1658	1666	1393	1736	2708	1664
AMPL133212	*SMO*	chr7:128845064-128845159	96	2958	2558	2581	2239	3385	2752	2007	2200	2182	2988	1582	1587	2519	2699	2115	2218	2813	1691	2026	1833	1775	2264	1567	2209	1346	3245	2441	2936	2553	3138	2735	3497	2395
AMPL122008	*SMO*	chr7:128845999-128846088	90	2010	1615	1748	1339	2227	1720	1219	1624	1203	2117	1157	1174	1705	1424	1002	1249	1335	1097	1498	915	1627	1897	1270	1810	1225	2654	2257	3085	2059	2065	2251	2668	1695
AMPL64101	*SMO*	chr7:128846361-128846416	56	816	580	680	583	794	712	428	530	367	736	536	342	615	387	370	400	441	416	632	221	522	554	495	750	466	1031	712	1167	540	491	829	772	591
AMPL482180	*SMO*	chr7:128850285-128850363	79	831	546	690	666	869	739	546	657	471	862	553	485	749	550	418	519	458	493	697	378	805	858	551	840	542	1268	1069	1600	901	911	1114	1039	740
AMPL492615	*SMO*	chr7:128851552-128851614	63	834	676	716	665	875	757	498	613	526	910	540	496	724	692	537	582	670	523	707	436	647	736	461	671	466	1084	909	1064	745	829	959	1098	708
AMPL304699	*BRAF*	chr7:140453120-140453223	104	1412	1228	1203	961	1559	1198	882	1072	929	1305	738	851	1052	1194	828	882	914	703	872	737	1020	1038	795	1158	794	1531	1209	1578	1250	1329	1195	1563	1093
AMPL1308445	*BRAF*	chr7:140481395-140481504	110	2172	2119	1955	1744	2571	2103	1574	1933	1829	2077	1099	1319	1939	2394	1634	1685	1933	1187	1395	1511	1380	1542	926	1365	1018	2160	1717	1998	1958	2119	1771	2330	1764
AMPL71577	*FGFR1*	chr8:38282155-38282216	62	1774	1469	1791	1275	1561	1727	1712	1247	1433	1204	1166	1278	1493	1871	848	1672	1535	1107	1497	1490	1567	1053	1656	1525	1337	2472	2365	1970	1746	1715	2085	2256	1591
AMPL71600	*FGFR1*	chr8:38285895-38285951	57	1657	1694	1792	1103	1571	1449	1481	1301	1398	899	1007	1000	1742	2700	1236	2048	1900	1049	1451	1989	1140	952	1262	1437	1307	1940	1815	1398	1333	1502	1577	2652	1524
AMPL74598	*JAK2*	chr9:5073732-5073791	60	898	1003	842	756	1331	1125	687	893	813	717	546	784	644	682	503	910	745	738	832	548	659	680	713	846	773	1494	1271	1189	1070	1179	1209	1554	895
AMPL483802	*CDKN2A*	chr9:21971131-21971219	89	1172	1465	1251	967	1187	1813	1234	1516	1270	396	784	1247	1125	530	887	1695	1617	1053	1333	1117	1063	1148	1190	1123	1282	2068	1802	2040	1698	2093	1581	2456	1350
AMPL73150	*ABL1*	chr9:133738311-133738379	69	1684	1664	1487	1409	2136	2006	1312	1627	1364	1730	1413	1428	1487	1185	1348	1413	1634	1181	1434	1325	1436	1328	1124	1636	1155	2229	1994	1796	1815	1417	1841	2600	1582
AMPL73075	*ABL1*	chr9:133747483-133747542	60	1082	1112	886	898	1263	1220	693	932	923	952	806	746	676	763	906	875	995	606	715	778	699	596	544	802	584	1215	852	802	837	624	843	1306	860
AMPL73104	*ABL1*	chr9:133748241-133748296	56	933	830	825	710	1008	922	708	870	782	880	903	890	780	579	634	793	748	638	816	539	1084	831	783	1123	894	1464	1503	1415	922	717	1264	1454	914
AMPL491413	*ABL1*	chr9:133748387-133748452	66	1035	913	866	767	1164	1169	703	858	716	994	880	846	738	590	722	831	983	728	788	777	971	863	745	1055	805	1575	1196	1273	1337	925	1131	1924	965
AMPL74743	*ABL1*	chr9:133750330-133750405	76	794	660	581	572	937	832	621	804	628	785	701	771	696	469	544	602	604	545	806	437	918	715	733	981	717	1336	1215	1258	981	718	1004	1224	787
AMPL482609	*NOTCH1*	chr9:139397712-139397791	80	1442	1317	1222	1130	1945	1562	1281	1209	1090	1480	1205	1210	1381	743	1035	913	1480	1090	1337	805	1716	1001	1020	1239	926	2042	1812	1409	1586	1344	1224	1995	1318
AMPL341576	*NOTCH1*	chr9:139399330-139399446	117	739	800	814	745	1368	1044	835	999	781	971	836	901	842	493	698	721	901	790	960	600	1612	864	919	1254	929	1836	1335	1596	1263	1433	1138	1418	1013
AMPL559669	*RET*	chr10:43609070-43609162	93	263	195	187	166	203	185	245	205	205	233	136	205	159	122	170	165	196	135	184	128	279	168	240	272	233	467	369	403	465	357	409	336	240
AMPL492612	*RET*	chr10:43609936-43610008	73	1429	1108	933	907	1121	1030	1168	1007	885	1183	1043	1038	1117	668	700	667	794	823	1159	588	1224	1049	1183	1355	1219	1968	1442	1946	1608	1161	1324	1819	1146
AMPL78961	*RET*	chr10:43613796-43613918	123	297	261	209	183	240	220	217	247	208	247	277	208	216	158	189	147	211	151	190	160	235	197	239	263	243	359	236	372	349	199	270	424	238
AMPL78994	*RET*	chr10:43615561-43615622	62	1474	1220	1063	1095	1305	1353	1227	933	960	1187	797	1104	955	756	951	801	1116	890	838	816	986	813	925	1155	1069	1827	1505	1388	1649	833	1382	1664	1126
AMPL79119	*RET*	chr10:43617404-43617456	53	1539	1239	927	811	1060	1197	1350	886	1118	1108	963	957	950	1393	1030	880	1167	847	980	935	926	795	958	892	847	1523	1179	1149	1532	1074	1154	2197	1111
AMPL81777	*PTEN*	chr10:89624241-89624298	58	77	69	42	41	64	54	80	46	74	80	69	70	77	91	29	65	75	56	57	44	62	37	36	28	32	112	99	87	67	115	76	74	65
AMPL464386	*PTEN*	chr10:89685262-89685356	95	775	439	338	353	391	499	853	525	763	463	384	443	394	823	573	592	535	693	650	901	473	305	493	460	429	695	571	731	671	804	660	714	575
AMPL80635	*PTEN*	chr10:89711882-89711932	51	461	400	251	248	264	361	639	484	487	462	337	441	444	706	502	651	614	508	509	630	375	344	485	441	372	740	682	679	734	995	768	559	518
AMPL82152	*PTEN*	chr10:89717581-89717639	59	1104	1074	818	958	994	1069	1034	751	719	917	705	635	712	770	704	794	939	752	887	943	937	546	718	598	569	1089	834	971	884	1136	892	1125	862
AMPL421278	*PTEN*	chr10:89717694-89717773	80	1761	1560	1461	1488	1834	1666	1981	1264	1316	1530	1164	1298	1206	1363	1049	1434	1529	1016	1280	1741	1396	1015	1171	978	1084	1774	1454	1633	1628	2207	1400	1871	1455
AMPL484297	*PTEN*	chr10:89720695-89720747	53	1045	1049	731	606	1016	963	1067	649	727	897	581	636	645	925	735	939	933	871	849	1054	955	566	681	618	667	1139	909	915	1034	1184	932	1421	873
AMPL416888	*PTEN*	chr10:89720795-89720884	90	879	802	596	616	868	945	949	589	653	761	360	462	488	713	560	699	811	635	762	825	699	431	579	528	533	926	801	833	851	1381	733	972	726
AMPL76043	*FGFR2*	chr10:123257996-123258043	48	868	748	727	660	798	847	709	584	571	741	683	682	569	798	529	559	655	585	619	260	765	496	666	783	664	1288	934	988	961	965	934	1071	741
AMPL76049	*FGFR2*	chr10:123274734-123274819	86	1721	1828	1280	1422	1538	1698	1747	1362	1388	1518	919	1111	1253	1634	1251	1249	1701	1063	1515	620	1443	812	1053	1253	1095	1878	1424	1657	1557	2052	1319	1652	1407
AMPL77536	*FGFR2*	chr10:123279481-123279543	63	1440	1687	1204	1378	1507	1643	1698	1318	1349	1598	791	1254	1070	2092	1320	1281	1645	1008	1265	644	1134	849	1001	1222	950	1798	1446	1459	1587	2179	1390	1914	1379
AMPL136501	*FGFR2*	chr10:123279630-123279717	88	2010	2069	1543	1633	1732	1919	1961	1656	1589	1876	1243	1830	1390	1882	1459	1553	2052	1226	1470	880	2074	1196	1507	1642	1403	2262	1906	2293	2605	2857	1715	2613	1783
AMPL120430	*HRAS*	chr11:533820-533912	93	1510	1620	1293	1325	1794	1617	1259	1484	1259	1503	774	1508	1221	950	1541	1726	1578	1125	1651	896	1626	1209	1176	1594	1191	2318	2129	1850	1960	2103	1608	1834	1507
AMPL84984	*HRAS*	chr11:534256-534309	54	810	805	763	616	750	741	670	826	699	944	576	880	887	672	784	1005	838	565	821	504	962	748	693	1022	1006	1595	1346	1123	980	1151	1013	1626	888
AMPL82662	*ATM*	chr11:108117818-108117865	48	502	539	370	348	425	540	460	478	605	511	473	506	441	528	515	493	571	496	503	430	394	401	492	526	462	644	551	557	466	564	536	858	506
AMPL82666	*ATM*	chr11:108119778-108119831	54	601	594	501	460	647	694	558	722	635	759	547	898	616	628	555	686	574	618	620	476	706	535	724	792	767	944	868	982	908	787	905	1233	704
AMPL82758	*ATM*	chr11:108123522-108123581	60	648	607	420	347	467	545	612	657	798	694	581	870	588	732	718	709	631	645	607	517	648	558	741	780	669	944	847	918	906	773	867	1105	692
AMPL82011	*ATM*	chr11:108137942-108138015	74	1108	1354	949	947	1159	1276	868	1019	1166	1062	912	1238	878	915	1014	908	1124	894	862	890	829	658	863	926	931	1392	1020	1084	1113	1175	1033	1782	1042
AMPL83554	*ATM*	chr11:108155126-108155179	54	858	962	697	896	1041	1097	838	801	943	988	773	1477	825	719	821	819	889	644	742	651	842	660	896	895	839	1260	1247	1100	1098	1060	1224	1549	942
AMPL83653	*ATM*	chr11:108170456-108170513	58	756	871	618	489	559	716	772	724	842	797	619	1084	687	677	637	752	730	607	679	457	667	563	827	843	777	1189	1088	976	939	1243	890	1132	788
AMPL83478	*ATM*	chr11:108172396-108172469	74	1225	1770	1288	1274	1604	1576	1204	1369	1326	1518	1463	2076	1386	1116	1157	1382	1353	1075	1305	990	1399	1046	1356	1366	1378	1916	1752	1671	1809	2409	1562	2599	1491
AMPL83779	*ATM*	chr11:108173613-108173666	54	434	520	384	347	456	575	422	488	518	555	449	524	426	528	493	486	516	425	487	375	428	328	474	492	385	608	559	586	509	722	524	892	497
AMPL379514	*ATM*	chr11:108180928-108180995	68	164	209	149	166	192	181	164	187	208	190	168	225	133	129	149	157	172	111	116	121	132	116	198	209	180	225	199	224	247	243	251	288	181
AMPL84622	*ATM*	chr11:108200917-108200966	50	465	429	315	354	394	505	391	492	581	424	597	610	463	633	522	509	621	541	416	375	340	311	419	459	419	667	490	535	564	609	597	796	495
AMPL143592	*ATM*	chr11:108204634-108204684	51	445	399	355	276	380	419	340	338	466	346	493	538	298	386	348	351	401	406	331	349	351	274	478	435	447	593	443	456	515	556	523	854	425
AMPL83501	*ATM*	chr11:108205763-108205828	66	841	922	1002	797	1006	1004	658	756	674	876	653	1330	639	564	638	729	786	775	864	489	882	592	885	896	876	1334	1195	1066	1113	1291	1062	1160	886
AMPL83530	*ATM*	chr11:108206582-108206636	55	643	870	729	674	848	882	633	719	648	827	928	991	760	575	553	690	775	637	790	384	733	602	877	811	782	1084	893	882	785	1137	951	1429	798
AMPL83902	*ATM*	chr11:108218064-108218138	75	52	92	41	73	71	89	69	54	76	77	74	91	75	105	84	77	123	44	54	46	40	57	59	43	39	209	249	172	171	277	208	109	97
AMPL85006	*ATM*	chr11:108225560-108225634	75	812	784	626	530	703	891	808	872	1125	832	608	959	628	928	886	847	831	814	782	844	635	583	734	838	703	1008	916	880	1073	1210	917	1219	838
AMPL84933	*ATM*	chr11:108236045-108236110	66	1329	1527	1193	1316	1573	1598	1094	1260	1515	1298	1230	1783	1152	1447	1317	1282	1489	1098	1221	1326	977	872	1040	1149	1104	1591	1223	1197	1447	1783	1265	2352	1345
AMPL85059	*ATM*	chr11:108236173-108236220	48	658	646	620	612	728	738	486	585	523	645	706	1100	596	431	479	573	600	573	664	377	755	645	833	891	940	1190	1135	1178	1025	1167	1139	1297	767
AMPL88109	*KRAS*	chr12:25378521-25378590	70	758	434	382	357	462	548	326	368	387	436	268	394	301	276	271	340	345	413	440	249	345	311	2176	496	418	648	562	617	587	412	440	582	480
AMPL87927	*KRAS*	chr12:25380258-25380338	81	1958	1328	1043	1112	1406	1239	950	1042	916	1111	563	1079	639	632	775	970	1181	848	991	818	916	815	4912	1030	957	1410	1548	1318	1405	1184	1071	1104	1196
AMPL553293	*KRAS*	chr12:25398237-25398305	69	723	505	379	428	488	479	358	379	406	422	317	468	363	354	300	357	425	352	386	314	388	362	2063	439	305	646	501	555	576	463	436	737	490
AMPL309408	*PTPN11*	chr12:112888142-112888228	87	2842	2142	1685	2035	2269	2192	1789	1575	1912	2095	1568	1678	1533	1890	1678	1608	2060	1380	1488	1530	1520	1328	1700	1856	1328	2250	2455	1976	2101	2667	1717	3536	1918
AMPL86500	*PTPN11*	chr12:112926836-112926896	61	2137	1690	1235	1479	1620	1561	1261	1370	1133	1531	1206	1048	1142	1154	1131	1208	1425	932	1097	1041	1183	1067	1239	1154	1001	1735	1646	1477	1371	1540	1397	2252	1358
AMPL87993	*HNF1A*	chr12:121431388-121431445	58	1304	782	849	762	1058	948	718	757	774	970	721	940	666	620	569	773	788	691	866	469	1165	883	1138	1206	987	1783	1806	1594	1246	1379	1288	1500	1000
AMPL507582	*HNF1A*	chr12:121432010-121432099	90	2227	1808	1410	1428	1827	1586	1357	1412	1411	1739	1125	1334	1079	1110	1220	1489	1998	1170	1580	1186	1525	1155	1438	1517	1247	2091	2124	1899	1936	2549	1570	2486	1595
AMPL87967	*FLT3*	chr13:28592607-28592663	57	1168	1330	1045	1273	1306	1352	962	1104	1034	3375	1142	1199	1280	1469	945	1849	1251	814	1029	819	1027	3305	1767	1142	744	1653	1253	1397	1125	1085	1183	2181	1363
AMPL89417	*FLT3*	chr13:28602324-28602384	61	1141	1333	1008	1122	1262	1225	901	971	882	3138	936	1144	1161	1252	842	1511	1037	832	1114	745	1014	3158	1747	1084	824	1627	1299	1336	1189	1207	1217	1780	1282
AMPL89439	*FLT3*	chr13:28608212-28608289	78	1355	1175	760	931	1136	1421	1153	1121	1365	3150	841	1063	1027	1829	1191	1869	1171	971	1159	1077	925	2958	1711	1154	805	1734	1288	1396	1448	1355	1264	2162	1374
AMPL90401	*FLT3*	chr13:28608332-28608386	55	1072	1039	755	799	915	1081	798	827	793	2551	844	801	855	1273	817	1313	906	848	979	658	722	2341	1498	920	675	1320	1008	1080	1041	793	1072	1665	1064
AMPL89861	*FLT3*	chr13:28610116-28610168	53	884	983	689	860	885	930	663	653	688	2472	748	801	843	895	677	1275	826	646	751	538	698	2053	1283	824	562	1324	967	1061	941	847	935	1306	953
AMPL91827	*RB1*	chr13:48919231-48919296	66	287	167	154	127	142	211	161	226	281	452	257	208	171	294	222	313	206	265	161	219	151	170	191	187	145	255	234	299	259	118	222	303	220
AMPL91829	*RB1*	chr13:48923136-48923198	63	125	174	92	109	106	132	95	84	114	233	71	69	97	178	100	136	154	86	65	69	52	55	57	56	27	276	334	253	217	270	269	125	134
AMPL255119	*RB1*	chr13:48942646-48942725	80	288	248	207	207	261	274	142	191	192	453	262	168	188	162	130	216	178	168	188	134	130	129	144	160	139	242	145	235	206	140	180	258	199
AMPL91909	*RB1*	chr13:48955530-48955603	74	1618	1440	1222	1489	1501	1662	1184	1244	1338	3384	992	1157	1178	1813	1188	1861	1364	922	1036	1148	1028	1177	959	1075	766	1414	1188	1311	1488	943	1210	2165	1358
AMPL416298	*RB1*	chr13:49027118-49027186	69	1293	1025	865	983	1048	1230	824	874	1043	2267	647	660	701	1406	971	1349	1057	834	762	883	670	715	680	774	582	1106	833	1047	957	740	837	1503	974
AMPL555556	*RB1*	chr13:49033827-49033903	77	1149	1253	902	1100	1134	1146	852	876	1021	2650	779	1000	966	1314	899	1491	1157	688	848	953	784	997	798	899	601	1198	1007	1170	1099	1004	865	1150	1055
AMPL147815	*RB1*	chr13:49037846-49037932	87	567	290	279	266	260	350	440	477	753	881	395	414	351	877	574	640	422	469	388	600	238	305	345	460	229	486	423	597	561	390	488	635	464
AMPL91174	*RB1*	chr13:49039149-49039216	68	1017	466	437	363	380	478	740	904	1168	1773	712	837	694	1392	878	1199	687	643	645	745	442	696	653	753	477	974	793	934	871	656	985	1162	799
AMPL329410	*AKT1*	chr14:105246406-105246502	97	1957	1960	1677	1582	2122	1924	1659	1474	1674	1967	1388	2011	1648	1657	2393	1953	1857	1432	1961	962	1783	1467	1365	2068	1529	2964	2298	2506	2392	2915	2158	2674	1918
AMPL90912	*CDH1*	chr16:68835616-68835684	69	2106	1845	1479	1878	1919	1760	1554	1578	1675	1818	1649	1834	1557	1387	1142	1449	1684	1107	1355	804	2076	1119	1260	1395	1487	2241	1754	1647	1431	1688	1654	2498	1620
AMPL91050	*CDH1*	chr16:68846080-68846148	69	1309	1226	965	1133	1193	1077	885	936	915	1188	1200	1240	1003	729	654	860	926	829	1021	391	1431	842	1009	1084	1182	1875	1394	1467	1077	1286	1142	1805	1102
AMPL91406	*CDH1*	chr16:68847263-68847322	60	1234	1178	856	1259	1041	1081	784	774	920	1055	1150	970	986	821	713	824	1122	759	854	482	1212	604	836	812	822	1406	1012	1096	835	966	940	1640	970
AMPL110679	*TP53*	chr17:7573997-7574069	73	1291	1371	1171	1148	1320	1352	1018	1139	1135	1039	857	1198	960	898	886	1083	1305	895	1194	845	1204	901	908	1236	955	1528	1498	1543	1558	1626	1324	2055	1201
AMPL117792	*TP53*	chr17:7577021-7577131	111	1978	1723	1820	1595	1956	1996	1756	1782	1752	1389	945	1806	1289	1119	1260	1521	1997	1431	1849	1386	1972	1352	1482	2095	1441	2176	2543	2645	2566	3142	2003	1955	1804
AMPL94170	*TP53*	chr17:7577522-7577591	70	1121	1288	1086	1086	1306	1327	1055	1154	1059	1173	655	1308	1132	943	848	1185	1380	905	1200	909	1308	998	962	1249	991	1545	1536	1503	1606	1860	1406	2414	1234
AMPL226789	*TP53*	chr17:7578180-7578288	109	1453	1448	1412	1140	1598	1460	1249	1482	1168	1144	1054	1505	1242	859	877	1173	1488	1130	1345	964	1645	1184	1192	1667	1330	1963	2075	2321	2182	2712	1690	2317	1483
AMPL226369	*TP53*	chr17:7578387-7578483	97	767	684	664	574	737	663	605	633	592	623	616	801	670	415	393	580	692	580	786	409	996	738	676	1044	784	1082	1258	1614	1076	1238	983	1378	792
AMPL658417	*TP53*	chr17:7578525-7578579	55	946	1062	947	844	953	1021	824	868	770	740	685	979	631	753	639	878	932	709	876	590	827	671	652	851	587	1069	1112	952	1031	1210	1080	1415	878
AMPL484618	*TP53*	chr17:7579345-7579445	101	1204	965	1064	887	1250	1050	1007	1110	1001	864	552	1152	702	585	683	998	948	853	1165	660	1336	871	938	1335	968	1642	1608	1753	1519	1777	1298	1057	1088
AMPL310032	*TP53*	chr17:7579859-7579941	83	1326	1152	1082	1019	1208	1181	1108	1185	1082	990	751	1014	777	720	668	972	1110	851	1240	790	1293	906	906	1287	971	1555	1518	1844	1334	1599	1217	1416	1127
AMPL484410	*ERBB2*	chr17:37880213-37880284	72	1328	977	969	866	1063	1102	853	738	943	1048	6932	918	3171	1138	1338	1048	1072	695	916	1088	1838	677	642	969	903	1459	1268	1153	1139	1337	946	1359	1309
AMPL93379	*ERBB2*	chr17:37880987-37881061	75	1339	1057	1021	939	1093	1137	848	908	841	1109	10198	1127	3738	887	1214	908	905	825	1114	760	2510	987	867	1377	1256	2124	1852	1815	1311	1566	1427	1647	1585
AMPL92464	*ERBB2*	chr17:37881297-37881364	68	1025	812	999	657	1004	818	774	772	779	889	8146	987	3859	876	943	709	640	606	775	629	1672	651	731	1293	1235	1546	1263	1497	1042	1553	1099	1227	1297
AMPL504796	*ERBB2*	chr17:37881433-37881497	65	1124	937	816	801	893	992	688	818	698	975	7348	807	3248	882	1066	922	836	637	950	819	1840	685	650	977	875	1496	1326	1182	1077	1034	1047	1406	1245
AMPL98573	*SMAD4*	chr18:48575133-48575188	56	333	222	188	170	119	264	271	361	429	323	331	349	246	392	348	411	480	310	313	288	196	227	446	305	215	449	483	495	489	374	515	516	339
AMPL98681	*SMAD4*	chr18:48581197-48581246	50	898	914	739	708	645	793	598	740	615	900	656	723	537	407	496	638	735	618	758	443	845	606	1300	720	687	1177	1221	1203	906	708	1080	1132	786
AMPL99638	*SMAD4*	chr18:48584511-48584567	57	[illegible]	[illegible]	[illegible]	[illegible]	[illegible]	[illegible]	[illegible]	[illegible]	[illegible]	[illegible]	[illegible]	[illegible]	[illegible]	[illegible]	[illegible]	[illegible]	[illegible]	[illegible]	[illegible]	[illegible]	[illegible]	[illegible]	[illegible]	[illegible]	[illegible]	[illegible]	[illegible]	[illegible]	[illegible]	[illegible]	[illegible]	[illegible]	[illegible]
AMPL552959	*SMAD4*	chr18:48586236-48586305	68	[illegible]	[illegible]	[illegible]	[illegible]	[illegible]	[illegible]	[illegible]	[illegible]	[illegible]	[illegible]	[illegible]	[illegible]	[illegible]	[illegible]	[illegible]	[illegible]	[illegible]	[illegible]	[illegible]	[illegible]	[illegible]	[illegible]	[illegible]	[illegible]	[illegible]	[illegible]	[illegible]	[illegible]	[illegible]	[illegible]	[illegible]	[illegible]	[illegible]
AMPL121852	*SMAD4*	chr18:48591825-48591924	100	[illegible]	[illegible]	[illegible]	[illegible]	[illegible]	[illegible]	[illegible]	[illegible]	[illegible]	[illegible]	[illegible]	[illegible]	[illegible]	[illegible]	[illegible]	[illegible]	[illegible]	[illegible]	[illegible]	[illegible]	[illegible]	[illegible]	[illegible]	[illegible]	[illegible]	[illegible]	[illegible]	[illegible]	[illegible]	[illegible]	[illegible]	[illegible]	[illegible]
AMPL163350	*SMAD4*	chr18:48593402-48593459	58	[illegible]	[illegible]	[illegible]	[illegible]	[illegible]	[illegible]	[illegible]	[illegible]	[illegible]	[illegible]	[illegible]	[illegible]	[illegible]	[illegible]	[illegible]	[illegible]	[illegible]	[illegible]	[illegible]	[illegible]	[illegible]	[illegible]	[illegible]	[illegible]	[illegible]	[illegible]	[illegible]	[illegible]	[illegible]	[illegible]	[illegible]	[illegible]	[illegible]
AMPL101246	*SMAD4*	chr18:48603031-48603113	83	[illegible]	[illegible]	[illegible]	[illegible]	[illegible]	[illegible]	[illegible]	[illegible]	[illegible]	[illegible]	[illegible]	[illegible]	[illegible]	[illegible]	[illegible]	[illegible]	[illegible]	[illegible]	[illegible]	[illegible]	[illegible]	[illegible]	[illegible]	[illegible]	[illegible]	[illegible]	[illegible]	[illegible]	[illegible]	[illegible]	[illegible]	[illegible]	[illegible]
AMPL116963	*SMAD4*	chr18:48604667-48604755	89	[illegible]	[illegible]	[illegible]	[illegible]	[illegible]	[illegible]	[illegible]	[illegible]	[illegible]	[illegible]	[illegible]	[illegible]	[illegible]	[illegible]	[illegible]	[illegible]	[illegible]	[illegible]	[illegible]	[illegible]	[illegible]	[illegible]	[illegible]	[illegible]	[illegible]	[illegible]	[illegible]	[illegible]	[illegible]	[illegible]	[illegible]	[illegible]	[illegible]
AMPL482833	*STK11*	chr19:1207000-1207098	99	[illegible]	[illegible]	[illegible]	[illegible]	[illegible]	[illegible]	[illegible]	[illegible]	[illegible]	[illegible]	[illegible]	[illegible]	[illegible]	[illegible]	[illegible]	[illegible]	[illegible]	[illegible]	[illegible]	[illegible]	[illegible]	[illegible]	[illegible]	[illegible]	[illegible]	[illegible]	[illegible]	[illegible]	[illegible]	[illegible]	[illegible]	[illegible]	[illegible]
AMPL503320	*STK11*	chr19:1220427-1220502	76	[illegible]	[illegible]	[illegible]	[illegible]	[illegible]	[illegible]	[illegible]	[illegible]	[illegible]	[illegible]	[illegible]	[illegible]	[illegible]	[illegible]	[illegible]	[illegible]	[illegible]	[illegible]	[illegible]	[illegible]	[illegible]	[illegible]	[illegible]	[illegible]	[illegible]	[illegible]	[illegible]	[illegible]	[illegible]	[illegible]	[illegible]	[illegible]	[illegible]
AMPL103879	*STK11*	chr19:1221236-1221320	85	[illegible]	[illegible]	[illegible]	[illegible]	[illegible]	[illegible]	[illegible]	[illegible]	[illegible]	[illegible]	[illegible]	[illegible]	[illegible]	[illegible]	[illegible]	[illegible]	[illegible]	[illegible]	[illegible]	[illegible]	[illegible]	[illegible]	[illegible]	[illegible]	[illegible]	[illegible]	[illegible]	[illegible]	[illegible]	[illegible]	[illegible]	[illegible]	[illegible]
AMPL490549	*STK11*	chr19:1223036-1223141	106	[illegible]	[illegible]	[illegible]	[illegible]	[illegible]	[illegible]	[illegible]	[illegible]	[illegible]	[illegible]	[illegible]	[illegible]	[illegible]	[illegible]	[illegible]	[illegible]	[illegible]	[illegible]	[illegible]	[illegible]	[illegible]	[illegible]	[illegible]	[illegible]	[illegible]	[illegible]	[illegible]	[illegible]	[illegible]	[illegible]	[illegible]	[illegible]	[illegible]
AMPL103227	*JAK3*	chr19:17945675-17945734	60	[illegible]	[illegible]	[illegible]	[illegible]	[illegible]	[illegible]	[illegible]	[illegible]	[illegible]	[illegible]	[illegible]	[illegible]	[illegible]	[illegible]	[illegible]	[illegible]	[illegible]	[illegible]	[illegible]	[illegible]	[illegible]	[illegible]	[illegible]	[illegible]	[illegible]	[illegible]	[illegible]	[illegible]	[illegible]	[illegible]	[illegible]	[illegible]	[illegible]
AMPL103630	*JAK3*	chr19:17947992-17948062	71	[illegible]	[illegible]	[illegible]	[illegible]	[illegible]	[illegible]	[illegible]	[illegible]	[illegible]	[illegible]	[illegible]	[illegible]	[illegible]	[illegible]	[illegible]	[illegible]	[illegible]	[illegible]	[illegible]	[illegible]	[illegible]	[illegible]	[illegible]	[illegible]	[illegible]	[illegible]	[illegible]	[illegible]	[illegible]	[illegible]	[illegible]	[illegible]	[illegible]
AMPL649396	*SRC*	chr20:36031712-36031771	60	[illegible]	[illegible]	[illegible]	[illegible]	[illegible]	[illegible]	[illegible]	[illegible]	[illegible]	[illegible]	[illegible]	[illegible]	[illegible]	[illegible]	[illegible]	[illegible]	[illegible]	[illegible]	[illegible]	[illegible]	[illegible]	[illegible]	[illegible]	[illegible]	[illegible]	[illegible]	[illegible]	[illegible]	[illegible]	[illegible]	[illegible]	[illegible]	[illegible]
AMPL104671	*GNAS*	chr20:57484403-57484471	69	[illegible]	[illegible]	[illegible]	[illegible]	[illegible]	[illegible]	[illegible]	[illegible]	[illegible]	[illegible]	[illegible]	[illegible]	[illegible]	[illegible]	[illegible]	[illegible]	[illegible]	[illegible]	[illegible]	[illegible]	[illegible]	[illegible]	[illegible]	[illegible]	[illegible]	[illegible]	[illegible]	[illegible]	[illegible]	[illegible]	[illegible]	[illegible]	[illegible]
AMPL104720	*SMARCB1*	chr22:24133965-24134014	50	[illegible]	[illegible]	[illegible]	[illegible]	[illegible]	[illegible]	[illegible]	[illegible]	[illegible]	[illegible]	[illegible]	[illegible]	[illegible]	[illegible]	[illegible]	[illegible]	[illegible]	[illegible]	[illegible]	[illegible]	[illegible]	[illegible]	[illegible]	[illegible]	[illegible]	[illegible]	[illegible]	[illegible]	[illegible]	[illegible]	[illegible]	[illegible]	[illegible]
AMPL107255	*SMARCB1*	chr22:24143229-24143298	70	[illegible]	[illegible]	[illegible]	[illegible]	[illegible]	[illegible]	[illegible]	[illegible]	[illegible]	[illegible]	[illegible]	[illegible]	[illegible]	[illegible]	[illegible]	[illegible]	[illegible]	[illegible]	[illegible]	[illegible]	[illegible]	[illegible]	[illegible]	[illegible]	[illegible]	[illegible]	[illegible]	[illegible]	[illegible]	[illegible]	[illegible]	[illegible]	[illegible]
AMPL106398	*SMARCB1*	chr22:24145525-24145589	65	[illegible]	[illegible]	[illegible]	[illegible]	[illegible]	[illegible]	[illegible]	[illegible]	[illegible]	[illegible]	[illegible]	[illegible]	[illegible]	[illegible]	[illegible]	[illegible]	[illegible]	[illegible]	[illegible]	[illegible]	[illegible]	[illegible]	[illegible]	[illegible]	[illegible]	[illegible]	[illegible]	[illegible]	[illegible]	[illegible]	[illegible]	[illegible]	[illegible]
AMPL484088	*SMARCB1*	chr22:24176335-24176402	68	[illegible]	[illegible]	[illegible]	[illegible]	[illegible]	[illegible]	[illegible]	[illegible]	[illegible]	[illegible]	[illegible]	[illegible]	[illegible]	[illegible]	[illegible]	[illegible]	[illegible]	[illegible]	[illegible]	[illegible]	[illegible]	[illegible]	[illegible]	[illegible]	[illegible]	[illegible]	[illegible]	[illegible]	[illegible]	[illegible]	[illegible]	[illegible]	[illegible]
