# Supplementary material for: Comparable genetic alteration profiles between gastric cancers with current and past Helicobacter pylori infection
Source: Sci Rep. 2021 Dec 6;11:23443. doi: 10.1038/s41598-021-02761-7 (PMC8648804; doi:10.1038/s41598-021-02761-7)
Supplement: Supplementary file 2 — Supplementary Information 2. [file 41598_2021_2761_MOESM2_ESM.pdf]

Comparable genetic alteration profiles between gastric cancers with current and past *Helicobacter pylori* infection

Sho Tsuyuki, Hideyuki Takeshima, Shigeki Sekine, Yukinori Yamagata, Takayuki Ando, Satoshi Yamashita, Shin Maeda, Takaki Yoshikawa, and Toshikazu Ushijima

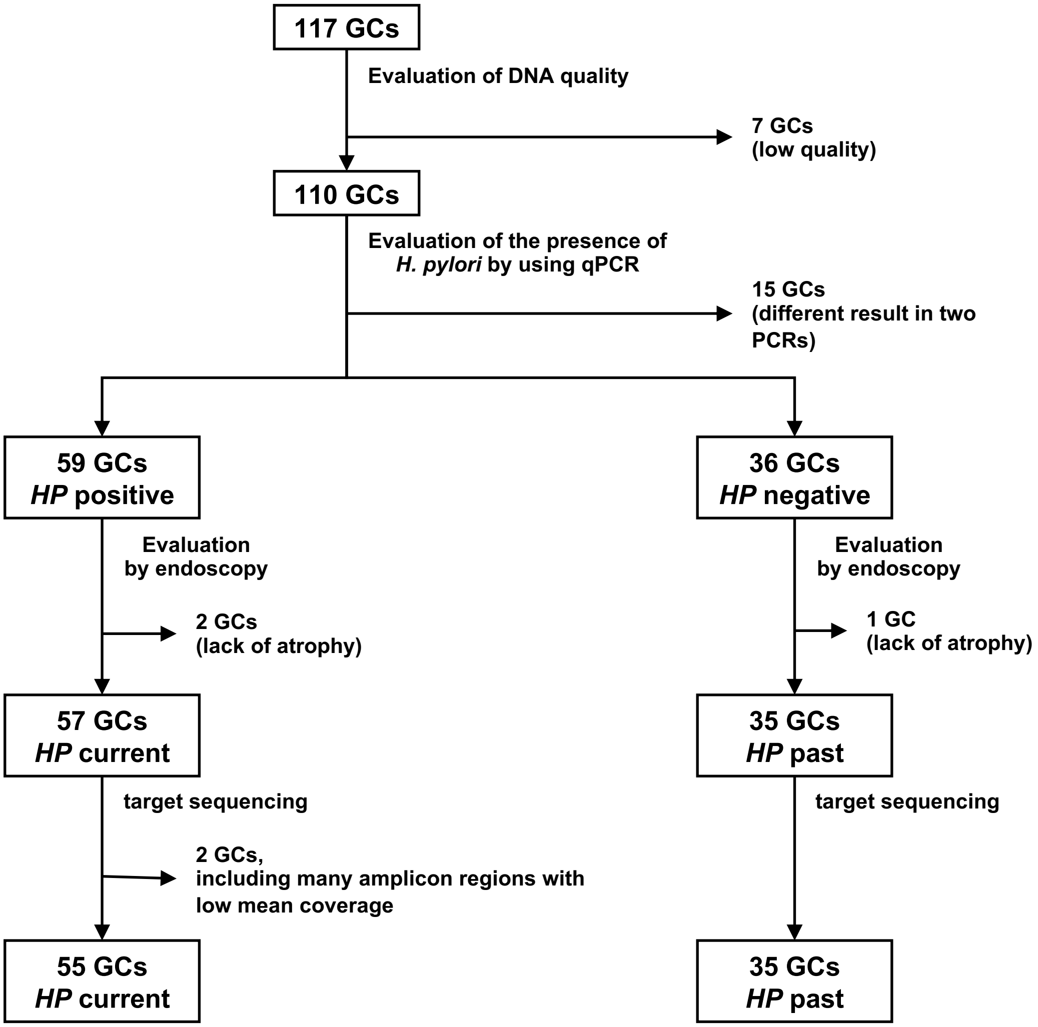

Supplementary Fig. S1

A flowchart of how 90 patients were selected from 117 patients.
